# Trade-offs and Noise Tolerance in Signal Detection by Genetic Circuits

**DOI:** 10.1371/journal.pone.0012314

**Published:** 2010-08-26

**Authors:** Raúl Guantes, Javier Estrada, Juan F. Poyatos

**Affiliations:** 1 Department of Condensed Matter Physics, Science Faculty, Universidad Autónoma de Madrid, Madrid, Spain; 2 Institute for Materials Science ‘Nicolás Cabrera’, Science Faculty, Universidad Autónoma de Madrid, Madrid, Spain; 3 Logic of Genomic Systems Laboratory, Spanish National Biotechnology Centre, Consejo Superior de Investigaciones Científicas (CSIC), Madrid, Spain; Fondazione Telethon, Italy

## Abstract

Genetic circuits can implement elaborated tasks of amplitude or frequency signal detection. What type of constraints could circuits experience in the performance of these tasks, and how are they affected by molecular noise? Here, we consider a simple detection process–a signal acting on a two-component module–to analyze these issues. We show that the presence of a feedback interaction in the detection module imposes a trade-off on amplitude and frequency detection, whose intensity depends on feedback strength. A direct interaction between the signal and the output species, in a type of feed-forward loop architecture, greatly modifies these trade-offs. Indeed, we observe that coherent feed-forward loops can act simultaneously as good frequency and amplitude noise-tolerant detectors. Alternatively, incoherent feed-forward loop structures can work as high-pass filters improving high frequency detection, and reaching noise tolerance by means of noise filtering. Analysis of experimental data from several specific coherent and incoherent feed-forward loops shows that these properties can be realized in a natural context. Overall, our results emphasize the limits imposed by circuit structure on its characteristic stimulus response, the functional plasticity of coherent feed-forward loops, and the seemingly paradoxical advantage of improving signal detection with noisy circuit components.

## Introduction

Signal transduction networks are commonly constituted by genetic circuits, or modules, comprised of a small number of interacting molecular elements. Recent accounts of how these modules compute biochemical information highlighted the intricate relationship between structure and function, and the capacity of these units to process different signal attributes (e.g., signal amplitude or frequency) [1]–[6].

This capacity is particularly relevant for cellular action. For instance, as part of the mating pheromone response, yeast uses a mitogen activated protein kinase (MAPK) module to sense different amplitudes of a signal, i.e., pheromone levels, in order to execute alternative developmental decisions [5]. The frequency of an oscillatory stimulus, such as the tumor necrosis factor-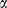 (TNF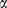) cytokine in inflamattory tissues [3], can equally play a fundamental role. The frequency of the TNF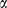 oscillation is read by the nuclear factor 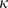
B signalling module, with different frequencies resulting in changes in timing and specificity in the transcriptional activation of downstream genes [6].

In fact, the relevance of oscillatory stimuli (and response oscillatory codes) to understand signaling systems is increasingly being appreciated. Oscillatory inputs can be used to probe and characterize genetic networks offering several advantages: the response may be easier to discriminate from noise than by applying a step stimulus [7], and systems identification theory [8] can be employed to validate molecular models of the network under study [9]–[12]. Alternatively, oscillations in protein abundance [2], [13], or protein localization [14], can act as a robust strategy to encode regulatory information, similar to the neural spiking codes.

**Figure 1:**
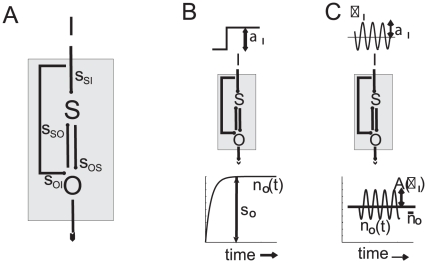
General scheme of signal detection by two-component modules. A. Illustration of the basic signal detection circuit studied. An input molecular species, I, acts as a biochemical signal on a minimal detection module (grey box) constituted by a sensor, S, and an output species, O. Lines indicate feasible interactions, either activation or inhibition, characterized by pairwise susceptibilities 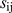 (autoregulations were also considered but not depicted for the sake of clarity). B. Signal amplitude detection: A sudden change in input (step signal) produces an amplitude change in the output concentration 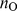 determined by the total output susceptibility 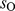. (C) Signal frequency detection: An oscillatory input signal of frequency 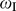 produces an oscillatory change in the output around the equilibrium value 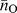, characterized by the relative amplitude 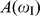.

Here, we study the response to an input signal (I) of a minimal genetic module –constituted by a sensor (S) and output (O) component (Figure 1A). We analyze the type of constraints that limit this unit to act as a multi-functional noise-tolerant sensing device (capable to read different attributes of a signal in the presence of biochemical noise [15]). Specifically, we ask which modules are more appropiate to process a particular signal feature, how these tasks are affected by noise, and which class(es) of circuits could then be functioning in a noise-tolerant manner.

To this aim, we used a unified theoretical description in terms of strength of interactions within module components (formally quantified as the gains or susceptibilities), and characteristic time scales (e.g., degradation rates) [16], [17]. Within a linear approximation for the dynamics, both the deterministic and stochastic response can be analyzed in terms of these quantities for static (long duration) and oscillatory signals. Our theoretical results are validated by numerical simulations of different detection modules, and the analysis of experimental data which suggests that gene circuits could exploit the properties discussed here under physiological stimulus conditions. As a whole, this study has implications for the design of synthetic circuits and the reverse engineering of natural ones.

## Results

### A framework to analyse amplitude and frequency detection in noiseless two-component circuits

The general three-node networks studied here are diagrammed in Figure 1A. All kind of interactions are allowed between sensor and output species (autoregulations are not shown for simplicity), and the input signal can act on both components, but there is no feedback to the input [18]. Interactions are characterized by their sign and strength, that we quantified with *pairwise susceptibilities*, 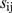, between network elements (defined below, see also Text S1 for details and derivations).

A genetic module works as a sensible detection device when it is capable of obtaining accurate information on changes of amplitude, 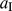, or frequency, 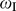, of the input signal, as schematically shown in Figs. 1B,C. To make this definition more quantitative, we employed two detection scores:

We used the *output susceptibility*, 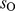, to estimate the potential of the module to detect amplitude variation (Figure 1B and Figure S1A) [16]–[18]. This measure quantifies the relative change in the output species at equilibrium, 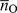, as the input signal changes –thus, the larger 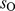, the better the detection–, and depends on the pairwise susceptibilities between module components (Figure 1A) as
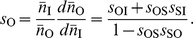
(1)Pairwise susceptibilities are expressed in terms of logarithmic gains or elasticities, i.e., 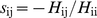, where 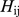 measures how the production/degradation balance of the 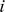
th component is affected by changes in the 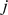
th one [17], [19].

To estimate frequency detection abilities, we considered a sinusoidal signal with amplitude 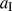 and frequency 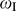 impinging on the module (Figure 1C). This signal takes the output from its initial equilibrium state (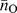) to an oscillating one [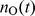] whose amplitude and phase-lag depend on the input frequency. A plausible measure of frequency detection is given by the range of frequencies 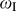 at which the amplitude of the oscillations are still distinguishable from the average equilibrium value, as usually quantified by the *output bandwidth*, 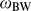. To formalize this idea, we first introduced the relative amplitude of the oscillatory output, i.e., 
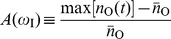
, and derived a linear approximation expression around 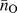 as (Text S1)
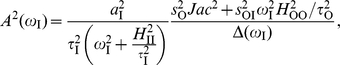
(2)where 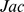 is the Jacobian determinant associated to local stability analysis of the steady state equilibrium, and 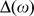 is defined by
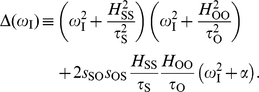
(3)Here, the corresponding elasticities are denoted by the 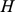
's, 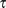
's are the decay rates of the molecular species, and 
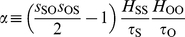
. Eq. (2) shows that the relative amplitude is the product of a low-pass filter induced by the signal –with bandwidth 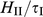
– times a second term dependent on susceptibilities and intrinsic time-scales of the module components. We numerically verified that this expression works well for oscillatory signals changing up to 50% the equilibrium value, Figure S1B. A measure of frequency detection is then given by the bandwidth of 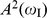, i.e., the range of frequencies where the amplitude of oscillations are above half its maximum value [8]. The larger 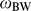, the more frequencies can be transmitted.

### Simple trade-offs emerge in noiseless signal detection

How does the specific module structure and biochemical parameters influence detection? The linear cascade, the simplest module in our general scheme, appears as a natural starting point to understand this issue. The output susceptibility is readily given in this case by the product of the corresponding pairwise susceptibilities (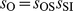), and the amplitude of the output to a sinusoidal signal by
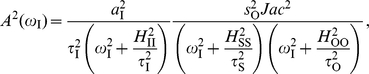
(4)i.e., the product of three low-pass filters, whose bandwidths are given uniquely by the lifetimes of each component.

Since bandwidth does not depend on susceptibilities, frequency detection can be adjusted independently of amplitude detection. Indeed, 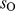 could be tuned to increase/decrease amplitude detection –by modifying pairwise susceptibilities –independent of lifetimes. It also became clear in this framework that the slowest time scale of the system determines its bandwidth (Figure S2A) [9], and that bandwidth is reduced by adding successive components to the cascade (Figure S2B). This implies that longer cascades buffer more efficiently transient stimuli, as it was recently tested in a synthetic genetic cascade [20].

More complex architectures are those showing feedback of the output component into the sensor species, i.e., 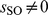. The output susceptibility reads then
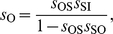
(5)with a negative (positive) feedback fulfilling 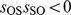 (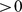). Additionally, the oscillatory output amplitude is similar to the linear cascade except for the second adding in 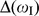, see Eqs. (2,3). For fixed degradation rates and no autoregulation of the module components (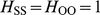), this term depends only on the product 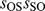, which we denoted as *feedback strength* (FS).

We used the previous analytic expressions to study detection in a collection of feedback modules with different biochemical parameters. To specifically characterize the role of FS, we fixed in this analysis both the input-sensor interaction (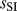) and the module time-scales (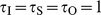), while allowing the feedback susceptibilities (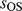 and 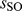) to vary within a range 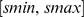 [18]: FSs are found in this way in the interval 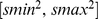 (positive feedback fulfilled FS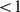 for the output steady state to be stable).

**Figure 2:**
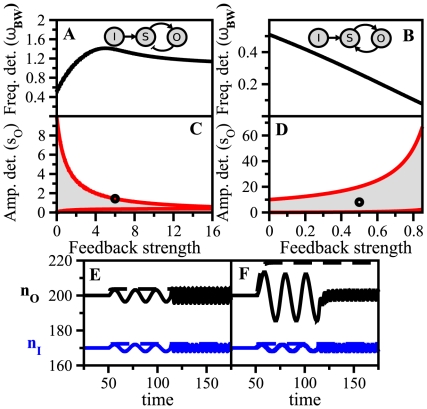
Amplitude/frequency detection in feedback circuits. Frequency detection (bandwidth 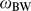, panels A, B) and amplitude detection (output susceptibility 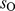, panels C, D) versus feedback strength FS for negative (left) and positive (right) feedback modules. The input/sensor susceptibility is fixed (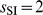) while the other pairwise susceptibilities change in the interval 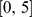. Time-scales are 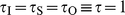. For each value of FS there corresponds a unique value of 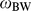 (black solid line in Figure 2 A,B) but a range of 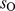
's (shaded region between red solid lines in Figure 2C,D). (E) Blue solid line: Input signal at two different frequencies, 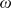 = 0.3 and 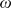 = 1.5 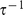, for a negative feedback circuit at FS = 6 and 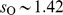 (black circle in Figure 2C). Black line: Output response. Blue and black dashed lines: signal pulse of the same amplitude as the oscillatory one, and the corresponding output response, respectively. (F) Response of a positive feedback circuit with FS = 0.5 and 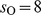 (black circle in Figure 2D) under the same signal and initial conditions.

In Figs. 2A–D, we plotted simultaneously bandwidth (frequency detection) and output susceptibility (amplitude detection) as a function of FS for negative and positive feedback, respectively –with FS = 0 corresponding to the linear cascade. Note that there exists a one-to-one correspondence between FS and 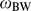 (black solid line in Figs. 2A,B) but a one-to-many for 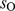 (shaded region in Figs. 2C,D). The same frequency detection can thus be achieved with different combinations of pairwise susceptibilities 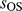 and 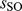, giving different 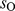 in Eq. (5). The maximum and minimum values for 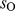 at each FS are plotted with red lines in Figs. 2C,D (specific limits given in Table S1). For positive feedback an increase in FS –producing a larger output susceptibility– entails a decrease in bandwidth, while for negative feedback the behavior is the opposite. This pattern clearly links FS –one fundamental design feature of architectures with feedback– with a trade-off between amplitude and frequency detection. This very same behavior is also observed in linear cascades with autoregulated components, as a function of autoregulation strength (Figure S3 and Table S1).

To further illustrate these trade-offs, we showed the dynamical response of each module to step and oscillatory input signals (Figures 2E–F, we used differential equations models of genetic circuits, Text S1 for details). We picked out intermediate FS values for both architectures (black circles in Figures 2C,D) and two different oscillation frequencies. We corroborated the analytical predictions of Figures 2A,B: for negative feedback the input signal induced a response of low amplitude but the oscillations were faithfully transmitted at high frequencies (Figure 2E). The positive feedback produced, on the other hand, a high amplitude response, but oscillations were poorly transmitted at high frequencies (Figure 2F).

### Feed–forward loops are flexible amplitude/frequency detectors

The presence of a direct interaction between input and output strongly modifies the detection characteristics just discussed. The best known architecture of this type, with 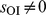, is of course the feed-forward loop (FFL) network motif [21]. Global susceptibility of the FFL module is a sum of direct and transmitted susceptibilities
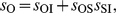
(6)and the amplitude of the oscillatory response is expressed from Eq. (2) as
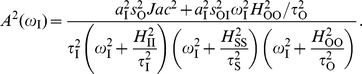
(7)The first term in this sum (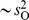) gives a low-pass filter identical to the linear cascade [Eq. (4)], while the second term (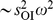) corresponds to a high-pass filter, produced by the direct action of the signal on the output. Frequency detection implies in this manner a competition between low- and high-pass filtering modes.

**Figure 3:**
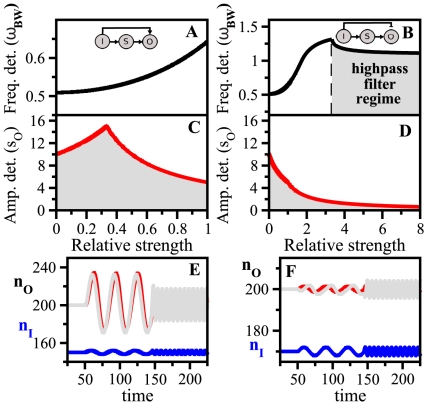
Amplitude/frequency detection in feed-forward loops. Frequency detection (bandwidth 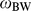, panels A, B) and amplitude detection (output susceptibility 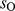, panels C, D) versus relative strength RS for coherent (left) and incoherent (right) feed-forward loop modules. Susceptibilities and time scales as in Figs. 2A–D. (E) Blue line: Input signal at two different frequencies, 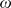 = 0.2 and 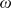 = 1 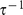. Grey line: Response of a coherent FFL module with RS = 0.33 and 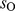 = 12 (larger than the maximum value allowed for a linear cascade, Figure 3C). Red line: Response of a positive feedback circuit with the same susceptibility (FS = 0.33). (F) Blue line: Input signal at 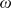 = 0.2 and 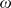 = 1 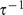 as in Figure 3E. Grey line: Response of an incoherent FFL module with 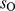 = 0.6 (RS = 6). Red line: Response of a negative feedback module with the same susceptibility (FS = 6).

Can we identify a single design attribute, like FS before, that allows a controlled comparison between FFL modules? We selected *relative strength* (RS; RS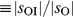) to be such determinant, since frequency detection behavior depends uniquely on this ratio. We started analyzing the most commonly found FFL motif, with all interactions/susceptibilities being positive [21]. In this case 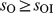 and low-pass filtering dominates. However, the high-pass contribution slightly enhances frequency detection with respect to the linear cascade (RS = 0). Thus, we observed a range of RS where amplitude detection increases above the maximum susceptibility exhibited in the linear cascade, due to the extra feed-forward connection, while frequency detection still improved (Figure 3A,C). This lack of trade-off when increasing amplitude detection contrasts to the positive feedback module architecture. We additionally exemplified this difference by computing the response dynamics of both circuits to an oscillatory input. In Figure 3E, we plotted the response of a coherent FFL (grey line) and a positive feedback (red line) to slow and fast oscillatory inputs (blue line). For slow oscillations, the amplitude of the oscillatory response appeared the same, since both architectures had equivalent total susceptibility. Faster oscillations were however better transmitted by the coherent FFL. Indeed, when we scanned for all possible modules able to outperform the linear cascade detection in amplitude and frequency simultaneously, we found that the coherent FFL was the only statistically significant motif (Figure S7A).

We next considered an incoherent FFL in which the feed-forward interaction is negative, whereas both 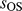 and 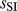 are positive [21]. The negative interaction reduced output susceptibility, Figure 3D, similar to the negative feedback module. In the same way, smaller 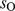
's implied larger bandwidth since the high-pass filter term became now more important. In the limit when the feed-forward arm was much stronger -in absolute value- than the indirect one (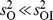), the incoherent FFL behaved as a band-pass filter (shaded region in Figure 3B, see also Figure S4), improving high frequency detection. To confirm this behavior, and also stress the differences with the negative feedback architecture, we plotted in Figure 3F the output trajectories of an incoherent FFL module (grey line) and a negative feedback module (red line) at the same susceptibility, with an oscillatory input (blue line). Here FS = RS = 6, so the incoherent FFL is in the high-pass filter regime (Figure 3B). While the response at slow frequencies is slightly better in the negative feedback, at high frequencies the incoherent FFL transmits oscillations with higher amplitude.

In sum, a FFL architecture achieves a flexible modulation of their detection properties by tuning the direct input-output interaction independently of the indirect ones.

### A framework to analyse amplitude and frequency detection in noisy two-component circuits

**Figure 4:**
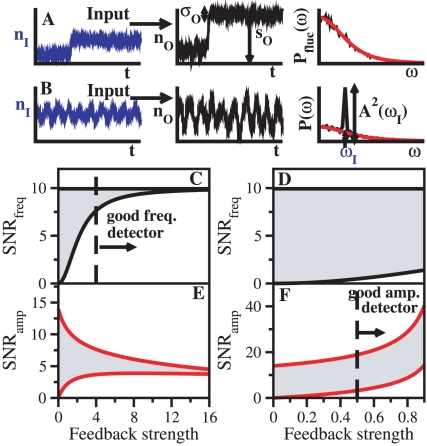
Quantifying signal detection with noisy components. A. Noisy amplitude detection. Left: A noisy step input acts on a detection module (also with intrinsic fluctuations). Middle: Output response characterized by susceptibility 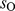 and amplitude of fluctuations around the equilibrium value, 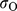. Right: Power spectrum of fluctuations around equilibrium. Black line is the numerical computation and red line is analytical result. (B) Noisy frequency detection. Left: Oscillatory input with noise. Middle: Output response in the time domain. Right: Output response in frequency domain [total power spectrum 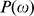]. The height of the peak at the input frequency 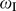 is proportional to the squared amplitude 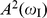. The theoretical background spectrum is plotted with a red line. (C) Frequency SNR for the negative feedback module as a function of FS. The dashed line approximately marks the FS value above which deterministic frequency detection is close to optimal for this module (Figure 2A). (D) Frequency SNR as a function of FS for the positive feedback module. (E) Amplitude SNR for negative feedback. (F) Amplitude SNR for positive feedback. The region of best amplitude detection performance (more than twice the linear cascade limit, Figure 2D) is approximately delimited by the dashed line. For panels C–F, susceptibility ranges and time-scales are as in Figs. 2,3A–D. Noise strength is determined by cell volume 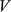, and here we take 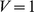 (Text S1).

A framework of noiseless signal detection does not always properly describe cellular information processing. The inherent stochasticity of biochemical reactions within cells [15] and signal intrinsic fluctuations (Figure 4A,B, left) may ultimately blur detection, mixing signal attributes with intrinsic and propagated fluctuations. This situation suggests the need for new measures to quantify sensible detection. We used signal-to-noise ratios (SNRs), broadly defined as the ratio between the intensity of output response over the noise (Text S1).

For amplitude detection, the SNR can be expressed as
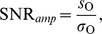
(8)where 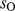 is the total output susceptibility (previously defined), and 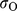 is the noise coefficient of variation (standard deviation of the fluctuations over the mean). This SNR reflects how detection of amplitude change may be corrupted by the relative output fluctuations (Figure 4A, middle, and Figure S5A).

For oscillatory signals, Figure 4B, output oscillations could of course be masked by fluctuations of large amplitude [22] but, more likely, detection will be limited by the frequency of output noise. The frequency content of the fluctuations in abundance around a mean value is given by its power spectrum denoted as 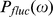 (Figure 4A, right). Analogously, for an oscillatory output (Figure 4B, middle) its total power spectrum, 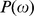, can be decomposed into a noisy background (red line in Figure 4B, left) and an oscillatory component at the input frequency (the peak in Figure 4B, right) [23]. The height of the oscillatory component is proportional to the amplitude of the output oscillations (Text S1).

We characterized the SNR for frequency detection as the ratio of average signal power to average noise power, both at the input frequency, which can be expressed as
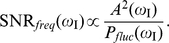
(9)where 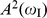 is the (relative) amplitude of the output oscillations at the input frequency, given by Eq. (2) within the linear approximation, and 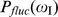 the power spectrum of the (relative) output fluctuations around the mean value.

Finally, we described both 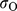 and 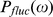 in terms of the same biological parameters used for the deterministic scores, i.e. susceptibilities, elasticities and degradation rates. This can be done using again linear approximations, leading to a fluctuation-dissipation theorem (FDT) for the covariance of the amplitude fluctuations [16], [18], [19]. The output noise amplitude around its equilibrium value can be decomposed in a sum of three contributions,
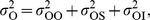
(10)where 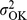 depends upon the intrinsic noise amplitude of species 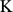 corrected by the interactions with the other species (Text S1 for detailed expressions and further discussion).

Moreover, 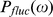 can be readily obtained from the FDT in the frequency domain, and also contains three contributions (Text S1),
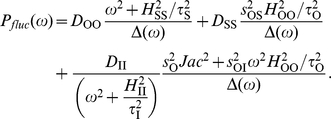
(11)


Each term in this sum depends on the noise strengths of its corresponding species (or diffusion coefficients 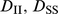 and 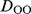, respectively, the rest of notation as before). The first two terms represent the contribution to the fluctuation spectrum of the intrinsic noise of output and sensor species –corrected by their feedback interactions. We denote this sum as module noise. The third term, the contribution of signal noise propagated through the network, is formally equivalent to the amplitude response to an oscillatory input, i.e., Eq. (2), since in the linear perturbation regime response is solely determined by intrinsic circuit features and the amplitude of the perturbation (irrespective of whether this is random or periodic). Using Gillespie simulations [24] we verified that the linear noise approximations Eqs. (10)–(11) quantitatively reproduce the numerical power spectra and coefficient of variation for different circuits (Figure 4A–B, left, and Figure S5A–B).

### Noise tolerance emerges in noisy signal detection

How is the amplitude/frequency detection performance changed in the presence of noise? We first plotted the amplitude/frequency SNRs for the negative (positive) feedback circuits as a function of FS (Figure 4C–F), as in the noiseless situation (Figure 2). In these analyses, we fixed input frequency (to the one where the amplitude of the oscillatory response was maximal) and also noise strengths, in order to compare on the same footing different module architectures. The behavior of 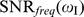 did not qualitatively change when selecting other input frequencies within the bandwidth of the noiseless circuit.

Two observations on frequency detection are relevant from inspection of Figures 4C,D: 1)there exists a range of potential 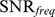
's for each FS [linked to the fact that the different terms in the spectrum, Eq. (11), are tuned by the individual susceptibilities], and 2)the maximum 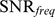 achievable, both for positive and negative feedback, cannot exceed the maximum value attained by the linear cascade (at FS = 0, see Text S1 for details). Interestingly, negative feedbacks working as good frequency detectors –large 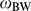
– exhibited also maximal 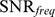, i.e., they are highly noise tolerant.

Regarding amplitude detection, it is clear that high output susceptibility might not only amplify signal but also fluctuations [18]. The latter, however, depends on the circuit class. For positive feedback, 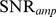 increases as a function of FS, i.e., fluctuations are less amplified than signal [18] (Figure 4F). For negative feedback, Figure 4E, we observed the opposite: a decrease in susceptibility (Figure 2C) is not followed by an effective noise reduction, and the overall 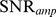 decreases. Note that in the regime where a positive feedback module functioned as a good amplitude detector doubling the linear cascade susceptibility (dashed line in Figure 4F, see also Figure 2), we observed a large 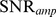. In summary, simple two-component modules performing a specific signal detection task in an optimal manner (good-detection regime) are tolerant to noise. When these very same modules work in poor-detection regimes, their performance also turned out to be more prone to noise corruption.

**Figure 5:**
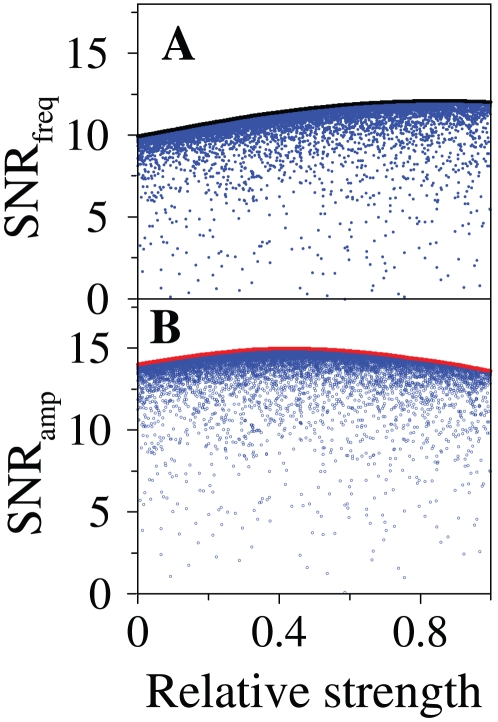
Noise tolerance is robust in coherent FFLs. A. Frequency SNR for the coherent FFL module. Black line: Maximum SNR. The scattered points correspond to an ensemble of 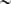
15,000 different circuits generated by randomly sampling susceptibility values within the interval 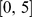. B. Amplitude SNR for coherent FFL. Red line: Maximum SNR. The scattered points correspond to the same ensemble of panel A.

What should we expect in a module acting as a good dual detector? We argued previously how the coherent FFL could simultaneously exhibit high susceptibility without losing bandwidth in a noiseless situation. How does noise affect these tasks? To answer this question, we plotted the SNRs as a function of relative strength in Figure 5 (compare also to Figure 3). We found that both 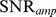 and 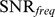 are able to surpass the linear cascade SNR limit (RS = 0). Moreover, this enhanced noise-tolerance is a robust feature, not subjected to a precise fine-tuning of the FFL parameters. For a uniform random sample of pairwise susceptibilities 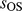 and 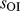 (scattered blue dots in Figure 5) most of the circuits are very close to the maximum SNRs. The reason for this behavior is that coherent FFLs are able to achieve high sensitivity due to the direct input/output interaction while reducing noise propagated through the sensor species (see discussion in Text S1 and Figure S5C,D). Scanning for all possible detection modules which could simultaneously improve amplitude and frequency SNRs, we found that a feed–forward loop type interaction was necessary to surpass the linear cascade limit (Figure S7B).

### Noise tolerance can be achieved by frequency filtering

An alternative strategy of noise tolerance might be at play in molecular circuits working as frequency detectors. In this situation, signals could be discriminated from fluctuations when their characteristic frequencies are different to the noise frequency content, i.e., the circuit effectively acts as a noise-filtering device. To determine the frequency range where noise is filtered out one can compare the corresponding bandwidth of the spectrum of fluctuations, Eq. (11), with that of the oscillatory amplitude, Eq. (2), with high-pass filtering mechanisms being at the core of noise tolerance (Text S1 and Figure S6).

**Figure 6:**
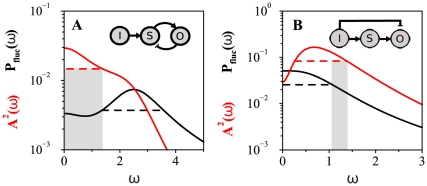
Noise frequency filters. Noise tolerance by filtering in frequency detection. A. Negative feedback module with 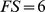 and susceptibility 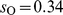. Red solid line: Amplitude of oscillatory output as a function of input frequency, 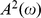. Red dashed line: Bandwidth of 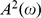. Black solid line: Power spectrum of fluctuations, 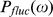. Black dashed line: Bandwidth of 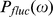. The grey region marks the noise-free frequency regime for the oscillatory response. In this case all the fluctuations are shifted to higher frequencies and do not overlap with the input frequencies detected by the module. B. Incoherent FFL with 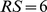 and 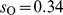. Lines are as in panel A. The grey region (noise-free frequency detection) is in this case only at high input frequencies.

We identified two potential high-pass filter contributions in the power spectrum [numerators 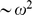 in Eq.(11)]. The first one appears in the intrinsic output fluctuations. This type of high-pass filtering works when the module exhibits a negative feedback interaction, 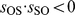, (Text S1 and Ref. [25]). Note that this term is not present in the amplitude of the oscillatory response, Eq. (2). Therefore, in the negative feedback circuit, one could expect that fluctuations are transmitted at higher frequencies than oscillations, allowing for a noise-free frequency regime. This is shown in Figure 6A. At moderate to high feedback strength (Figure S6), there are negative feedback circuits whose fluctuation bandwidth (dashed black line in Figure 6A) is always at higher frequencies than the oscillatory bandwidth (red dashed line in Figure 6A) allowing for a noise-free frequency regime in transmitted oscillations (grey shaded region in Figure 6A).

A second high-pass filter appeared when describing the propagated fluctuations from the signal to the output [last term in Eq. (11)], i.e., in a FFL architecture. This filter is also part of the oscillatory response, and dominates in the incoherent FFL (Figure 3B). However, the two additional terms in Eq. (11) due to intrinsic circuit fluctuations are low-pass, compensating in part the shift to high frequency noise. Thus, incoherent FFLs may act as noise-tolerant systems when transmiting high frequency oscillations. This is demonstrated in Figure 6B. In the case that the direct susceptibility 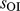 is larger than the global susceptibility 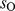 (i.e., RS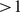, which is only possible in the incoherent FFL), the high-pass filter for the transmitted oscillations dominates. In this situation, there is a range of high frequency oscillations free of noise (grey shaded region in Figure 6B). The bandwidth of the fluctuation spectrum is however still large, i.e., no perfect filter exists unlike the negative feedback case.

These mechanisms of noise filtering would not be active in the limit when the detector module lacked intrinsic noise, 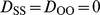, and thus both signal and noise frequencies were transmitted in the same way [compare Eqs. (2) and (11)]. In this case, the SNR depends only on the input oscillatory amplitude 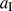 and noise intensity 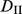 as 
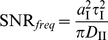
 (independent of 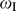). Thus, intrinsic noise in both output and sensor species emerged as a fundamental condition to alter spectral properties of the output fluctuations inducing noise tolerance.

### Signal detection properties in genetic circuits regulating sugar metabolism

Kaplan *et al.* [26], [27] recently measured the *in vivo* production rate (input function) of different sugar utilization genes in *Escherichia coli*, as a function of two different inputs: cAMP, which activates in a graded manner CRP, one of the master transcription factors in *E. coli* [26], and the cognate sugar. CRP activates most of the genes involved in sugar metabolism by means of a coherent FFL architecture, through the activation of an intermediate transcription factor [26]. The exception is the galactose utilization system which forms an incoherent FFL and shows a non-monotonic input function [27]. High resolution experimental measurements of production rates were made by spanning the whole range of physiological response for both inputs. We then considered these systems as good candidates to qualitatively assess if the signal detection features presented here can be found in living cells under natural conditions. We used simple mathematical models, fitted to the experimental input functions, to obtain the detection properties of these circuits with the preceding theory (Ref. [27] and Text S1).

**Figure 7:**
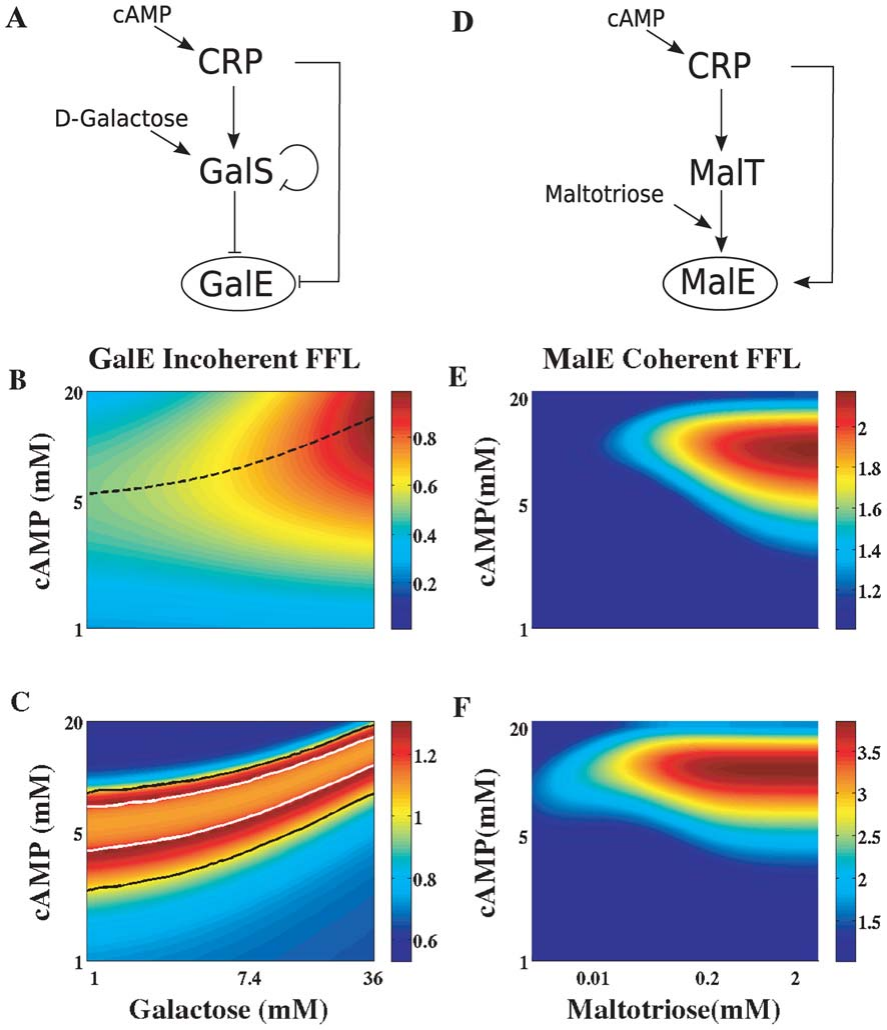
Response features of natural FFLs. Detection properties of FFL circuits involved in sugar metabolism. A. The *galETK* operon response to cAMP and galactose is mediated by an incoherent FFL interaction, where the external inducers cAMP and D-galactose are required for CRP and GalS activity, respectively. D. The *malEFG* operon is activated by cAMP and maltotriose through a coherent FFL involving CRP and MalT transcription factors [26]. B. Production rate of the *galE-* 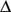 *galR* system. A mathematical model was fitted to the experimental input function (Ref. [27] and Text S1). For each galactose concentration, the production rate reaches a maximum at a particular value of external cAMP (shown with the dashed black line). C. Oscillatory bandwidth of the *galE-* 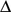 *galR* incoherent FFL for the same input ranges. White lines delimit the cAMP boundaries where the system acts as a band-pass *frequency* filter. Black lines mark the boundaries of partial noise filtering (as illustrated in Figure 6B). E–F. Detection in the presence of noise for the CRP/MalT/MalE coherent FFL. We fitted a simple mathematical model to the experimental input functions (Text S1) to calculate the SNR for both amplitude and frequency detection as a function of both inputs. E. Amplitude SNR normalized by the maximum SNR obtained setting the direct cAMP/malE interaction to zero (linear cascade limit). F. Frequency SNR normalized by the linear cascade limit.

We focused first on the incoherent FFL case, Figure 7A–C. For a fixed value of the sugar input, the cAMP/CRP response shows a maximum (Figure 7B and Ref. [27]) indicating that the galactose system operates as a band–pass detector for cAMP signal amplitude. This amplitude filter behavior was also observed in synthetic incoherent FFL circuits in *E. coli* [28], [29]. The reason behind the band–pass feature is that the susceptibility of the output (in this case, GalE protein) changes sign, since the direct CRP/GalE activation saturates and the negative interaction dominates, repressing the galE promoter at high cAMP levels (Figure S8). Therefore the relative strength is high around the maximum of the input function, and we expect band–pass filtering also for oscillatory signals. This is shown in Figure 7C, where the bandwidth of the galE response for an oscillatory cAMP input is plotted in a color code as a function of cAMP and galactose concentrations. White lines delineate the cAMP boundaries, for a fixed galactose concentration, where the system behaves as a band–pass frequency detector. Moreover, taking into account the noise in biochemical reactions, this system is also able to filter fluctuations for high–frequency oscillations by the mechanism discussed in the previous Section (noise filter range is marked with black solid lines in Figure 7C). We also analyzed a synthetic band amplitude detector constructed by Basu *et al.* [28], using parameters estimated from experimental data. Band detection is observed in this case as a function of a single input (AHL) but we reached identical conclusions: band–pass frequency and noise filtering mechanisms operate in the regime of band amplitude detection (Figure S9) and are thus intrinsic properties of incoherent FFLs.

As a case study of a natural coherent FFL, we chose the maltose regulon, Figure 7B. This is one of the simplest systems investigated in [26] since no additional autoregulations are present in the FFL components, and the inducer maltotriose acts postranscriptionally (favouring MalT activation by self-association [30]). Fitting the production rates of both MalT and MalE to the experimental input functions we set out a concise mathematical model for the malE response in terms of both inducers and the sensor transcription factor MalT (Text S1). When comparing with the linear cascade limit (neglecting the direct CRP/MalE interaction) we corroborated that bandwidth and susceptibility of the coherent FFL are larger in the whole input range. One of our main findings was that, for moderate input/sensor strengths, a coherent FFL was capable of improving signal detection in the presence of noise, giving SNRs beyond the linear cascade limit. We plotted the SNR in amplitude (Figure 7E) and frequency (Figure 7F) divided by the maximum value achieved by a linear cascade module in the whole input range (keeping identical susceptibilities for CRP/MalT and MalT/MalE interactions). As seen from Figure 7E–F, the SNR can improve up to factor of two (in amplitude) or three (in frequency)

## Discussion

We introduced an analytical framework to study the amplitude and frequency response of a general class of two-component genetic circuits (Figure 1). Signal sensitivity was quantified in terms of susceptibility (for amplitude detection) or bandwidth(frequency detection). In the simplest scenario, the linear cascade I
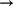
S
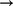
O, we found that these responses act independently (constraint-free), and that the slowest time scale of the cascade [9], and also its length [20], were limiting factors on maximal frequency transmission (i.e., bandwith).

For circuits with additional interactions, we found that frequency detection was dependent on a single parameter biologically meaningful(feedback strength, autoregulation strength, or relative strength in the case of feed–forward loops). In this way, we could show that feedback of the output species back to the sensor, while improving amplitude or frequency detection in comparison to the linear cascade, manifested the presence of functional trade-offs. Indeed, optimizing circuit design for amplitude detection (positive feedbacks with large FS) reduced frequency detection capacity. Alternatively, an optimized frequency detector (negative feedbacks with large FS) can hardly detect amplitude. These trade-offs similarly applied to linear cascades with autoregulatory loops.

However, direct action of the signal on the output species, in a FFL architecture, modifies these features. We showed how the coherent FFL improved both amplitude and frequency detection, and the potential of incoherent FFLs to work as high-pass frequency filters. Previous theoretical [31] and experimental studies [27]–[29] demonstrated that incoherent FFLs could also act as band-pass detectors in signal amplitude, providing maximal output activity at intermediate signal levels. The ability of incoherent FFLs to respond to high frequency time-periodic stimulation has been also noticed [32] using boolean regulatory functions and trains of pulses. We showed here how band-detection in frequency is possible for a large enough ratio of direct and indirect susceptibilities (relative strength). This critical parameter links in this way band-pass response for both static and oscillating signals. These features make FFL architectures the best design to achieve flexible signal detection, at the cost of a decrease in amplitude/frequency detection when compared to circuits honed in to these tasks, i.e., cascades with feedbacks. A similar methodology to the one employed here (systematic sampling of three node networks and linear approximations) but disregarding noise effects, has been recently used to show that incoherent FFLs and negative feedbacks were the only two robust topologies achieving biochemical adaptation [33].

To consider scenarios where biomolecular noise could be relevant [15], we used two measures of detection based on signal-to-noise ratios. Using this formalism, we argued how optimal frequency or amplitude detectors (feedbacks with large FS) were also particularly noise tolerant. Indeed, positive feedbacks (or autoregulation) were previously revealed to improve amplitude detection, while minimizing signal propagated noise [18] (see Figure 4F, where intrinsic noise was also included).

Interestingly, module noise [first two terms in Eqs. (10)–(11)] can enhance both amplitude and frequency SNR relative to the linear cascade in some architectures. This is the case of coherent FFLs. In this type of circuits, the direct input/output interaction increases signal sensitivity and simultaneously reduces propagated noise through the sensor component, eventually allowing a reduction of the total output noise. This improvement of SNR in coherent FFLs could not be found if only signal noise is taken into account [18].

Can noise tolerance be achieved in other ways? We discussed an alternative based on noise frequency filtering. In a negative feedback the module noise can be in the high frequency regime [25] but the signal propagated noise (and thus the propagated periodic signal) be in a lower frequency regime. This allows an effective separation of time scales for oscillations and fluctuations: fluctuations can be faster than the transmitted oscillation frequencies.

Notably, incoherent FFLs were found to exhibit the opposite behavior. For these modules, signal fluctuations are accelerated due to the direct interaction between input and output, and thus the module is also responsive at high periodic frequencies, but intrinsic fluctuations may be much slower. Thus incoherent FFLs can separate oscillation and fluctuation time scales only at high oscillatory frequencies. These results emphasize the complexity of analyzing noise even in simple scenarios, where all possible noise sources could exhibit counfounding effects (see [34] for another example, where noise reduction in a molecular species with negative feedback may be at the expense of increasing noise in the other species).

To evaluate some of the discussed signal-detection properties in specific systems, we obtained the response of several FFLs under natural conditions by fitting model circuit parameters to experimentally measured data. We confirmed in this way the possibility of band-pass filtering and noise-tolerance in the natural incoherent FFL associate to the GalETK operon (Figure 7A–C). These features were also corroborated with data from a synthetic incoherent FFL assembled with the LuxR, CI and LacI transcription factors [28]. Moreover, we also verified that a coherent FFL associated to maltose metabolism (Figure 7D–F) could exhibit –in the natural range of the corresponding stimuli– better amplitude and frequency detection than a linear cascade, when biochemical noise was also taken into account.

The dynamic features of noise can also be used to extract information about the relevant interactions and strengths of simple genetic circuits [35]–[37]. In this sense, our work may be useful in reverse engineering contexts: measuring frequency transmission of oscillatory signals is feasible but technically difficult [9], [10], because the input should be tightly controlled in some kind of microfluidic device. Alternatively, using standard single cell techniques one could measure a long enough time series of the fluctuations around steady state in the absence and presence of a permanent signal to obtain the corresponding spectra [36], [38], and subtract them to compute the contribution of the propagated noise from the signal [third term in Eq. (11)]. This contribution gives the same information about circuit parameters than the oscillatory response as a function of input frequency, but can be obtained with less experimental effort.

## Methods

Detailed derivations of theoretical expressions used in the paper, models, numerical simulations and fittings to experimental data are provided in Text S1.

## Supporting Information

Text S1 Detailed mathematical derivations, additional analyses and discussions, numerical models and tests of approximations used in the main text and model fitting to experimental data.
(0.22 MB PDF)

Figure S1 Response properties of a linear genetic cascade. A. Output relative change after a step input signal [Eq. (1.26) in Text S1], as a function of susceptibility. Black circles: a_I_ = 0.01. Red circles: a_I_ = 0.1. Solid lines are the linear predictions given by Eq. (1.27) in Text S1. B. Squared relative amplitude of the output oscillatory response to a signal [Eq. (1.8) in Text S1], as a function of the signal frequency ω_I_. Black circles: a_I_ = 0.01. Red circles: a_I_ = 0.1. Blue circles: a_I_ = 0.5. Solid lines are the theoretical predictions given by Eqs. (2–3) in main text. Kinetic equations and additional parameters are provided in Section 6 of Text S1.
(0.03 MB EPS)

Figure S2 Dependence of the frequency response of the linear cascade on time scale and cascade length. A. Black solid line: Squared amplitude as a function of input frequency for a three tier cascade (Input-Sensor-Output) with degradation rates δ_I_ = δ_S_ = δ_O_ = 1. Red line: δ_I_ = δ_S_ = δ_O_ = 2. Blue line: δ_I_ = 1, δ_S_ = δ_O_ = 2. Other parameters are input amplitude a_I_ = 0.1 and output susceptibility s_O_ = 4. B. Dot-dashed line: Squared amplitude of oscillatory response for a single species with periodically forced production rate. Dashed line: Response for a two-layered cascade (oscillatory input acting on a single component). Solid line: Three layer cascade with oscillatory input acting through an intermediate sensor species. Parameters are δ_I_ = δ_S_ = δ_O_ = 1, a_I_ = 0.1 and s_O_ = 4. The shaded region shows the bandwidth of the single species response, which is equal to the degradation rate δ_O_.
(0.04 MB EPS)

Figure S3 Amplitude/frequency detection for modules with output autoregulation as a function of autoregulation strength (ARS, defined in Text S1). A,C: Negative autoregulation. B,D: Positive autoregulation. A. Bandwidth for negative autoregulation of the output component. C. Susceptibility for negative autoregulation. Red line is the maximum output susceptibility as a function of ARS (Table S1) and grey shaded region the range of possible susceptibilities for individual interactions in the interval [0,5]. B. Bandwidth for positive autoregulation of the output element. D. Output susceptibility for positive autoregulation. Red lines and grey region as in panel C.
(0.29 MB EPS)

Figure S4 High-pass filtering behavior of an incoherent FFL. Grey line: Theoretical oscillatory amplitude as a function of input frequency for an incoherent FFL module with s_O_ = 0.6 and RS = 6. Grey circles are the numerical results. Black line: theoretical oscillatory amplitude for a negative feedback with the same s_O_ and FS = 6. Black circles: numerical results. Input amplitude is a_I_ = 0.013. Red dashed lines indicate the input frequencies for the trajectories shown in Fig. 3F in main text. See Section 6 in Text S1 for model details.
(0.03 MB EPS)

Figure S5 Noise properties of FFL circuits. A. Output noise (coefficient of variation) as a function of input noise tuned by means of the “volume” factor V_I_. Solid lines represent theory while circles denote numerical simulations. Red: linear cascade. Blue: coherent FFL. Green: incoherent FFL. Parameters: s_SI_ = 1, s_O_ = 2, V_S_ = V_O_ = 1, and τ_I_ = τ_S_ = τ_O_ = 1. The relative strength is RS = 0.5 both for coherent and incoherent FFLs. B. Power spectrum of the output response to an oscillatory signal of frequency ω_I_ = 1 and amplitude a_I_ = 0.05 for the same circuits. Parameters as in panel A, except V_I_ = 2 for all circuits, and RS = 1 for the incoherent FFL. Black lines: numerical simulations. Colored lines: theoretical background spectra. Red: linear cascade. Blue: coherent FFL. Green: incoherent FFL. Crosses mark the peak height at the signal frequency for each genetic circuit. Inset: Numerical peak heights as a function of input frequency (black circles). Red and green lines correspond to the theoretical amplitudes A^2^(ω_I_), Eq. (2) in main text, for the linear cascade and incoherent FFL, respectively. C. Output coefficient of variation as a function of output susceptibility [from Eq. (2.44) in Text S1] at s_SI_ = 2 for a linear cascade (red) and a coherent FFL (blue). Time scales are τ_I_ = τ_S_ = τ_O_ = 1, “system size” factor V = 100 for all components (with equilibrium values I^eq^ = S^eq^ = O^eq^ = 1) and the rest of the interactions change in the interval [0,5] as in main text. D. Amplitude of maximum oscillatory response versus fluctuation power spectrum at the same frequency, for the same sampling of circuits shown in panel C.
(2.23 MB EPS)

Figure S6 Noise frequency filtering range in negative feedback and incoherent FFLs. Filter range, Eq. (4.69) in Text S1, for negative feedback circuits (A) and incoherent FFL circuits (B) as a function of feedback and relative strength, respectively. The shaded region in panel A marks the regime where fluctuations are completely filtered out (FS>5), since they are shifted at frequencies higher than the oscillatory response bandwidth.
(0.20 MB EPS)

Figure S7 FFL interactions are required for optimal amplitude/frequency detection. A. Relative frequency of two-component detection motifs simultaneoulsy improving amplitude/frequency detection in a noiseless situation (both susceptibility and bandwidth larger than the linear cascade limit). B. Relative frequencies of two–component detection modules with both amplitude and frequency signal-to-noise rations larger than the linear cascade limits. CFFL: Coherent FFL. IFFL: Incoherent FFL. PF: Positive feedback. NF: Negative feedback. PAR: Positive autoregulation. NAR: Negative autoregulation. F: Feedback (it can be either positive or negative). MIX: Combination of at least two interactions (feedback or autoregulations) with different sign. See Section 5 in Text S1 for statistical estimations.
(0.01 MB EPS)

Figure S8 Band frequency filtering in the GalS/GalE system. GalE production rate and susceptibilities as a function of external cAMP concentration for [galactose] = 6 mM. The maximum in production (marked with a cross in Figure S8A) coincides with the change of sign of the output(GalE) susceptibility (red line in Figure S8B). GalS/GalE and CRP/GalE susceptibilities are shown with green and black dashed lines respectively in panel B. See Section 7 in Text S1 for model details.
(0.02 MB EPS)

Figure S9 Band–pass frequency filtering of a quorum sensing network. A. The synthetic construction experimentally studied by Basu et al., Ref. [28] in main text, includes an incoherent FFL where the input AHL/LuxR activates both CI and LacI repressors, while CI also shuts off LacI expression. The output of the system is monitored by a LacI dependent GFP. B. GFP output measured as a function of external AHL. The inset shows a zoom of the GFP response around the peak, where the oscillatory high–pass filter and noise filtering regimes are indicated by arrows. C. LacI susceptibility as a function of AHL. Note that it changes sign around maximum GFP response. D. GFP oscillatory response(green line) for a periodic AHL input (blue line) with mean concentration 0.04 µM (orange circle in the inset of panel A). Model details are given in Section 7 of Text S1.
(0.12 MB EPS)

Table S1 Definition of frequency detection parameters and corresponding susceptibility ranges for simple network motifs.
(0.03 MB PDF)
