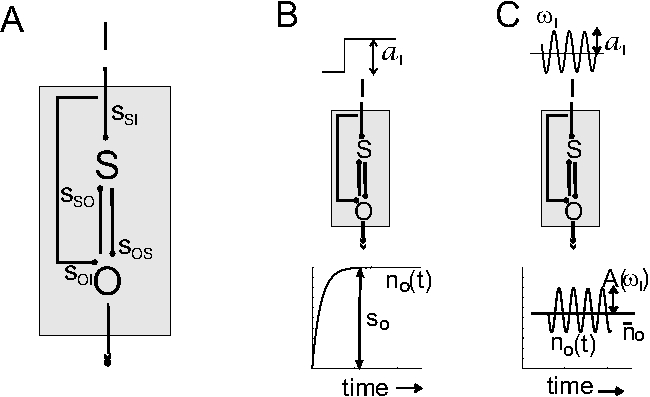# Correction: Trade-offs and Noise Tolerance in Signal Detection by Genetic Circuits

**DOI:** 10.1371/annotation/1c817cc7-4fb8-4fd5-a9c6-04b8d0e6c3fb

**Published:** 2010-09-30

**Authors:** Raúl Guantes, Javier Estrada, Juan F. Poyatos

Some text in Figure 1 was incorrectly replaced by symbols. Please view the correct figure here: 

**Figure:**