# Supplementary material for: Trade-offs and Noise Tolerance in Signal Detection by Genetic Circuits
Source: PLoS One. 2010 Aug 26;5(8):e12314. doi: 10.1371/journal.pone.0012314 (PMC2928721; doi:10.1371/journal.pone.0012314)
Supplement: Text S1 — Detailed mathematical derivations, additional analyses and discussions, numerical models and tests of approximations used in the main text and model fitting to experimental data. (0.22 MB PDF) [file pone.0012314.s001.pdf]

Trade-offs and noise-tolerance in signal detection by genetic circuits

Raúl Guantes[†], Javier Estrada[†], Juan F. Poyatos[‡]

[†] Department of Condensed Matter Physics, Facultad de Ciencias, Universidad Autónoma de Madrid, Madrid, Spain, and Instituto 'Nicolás Cabrera', Facultad de Ciencias, Universidad Autónoma de Madrid, 28049 Madrid, Spain
[‡] Logic of Genomic Systems Laboratory, Spanish National Biotechnology Centre, Consejo Superior de Investigaciones Científicas (CSIC), Madrid, Spain

Contents

1 Response of deterministic two-component networks to constant and oscillatory signals 2
1.1 Frequency detection properties of a linear cascade 6
1.2 Simultaneous determination of bandwidth and susceptibility for two-component networks 8

2 Amplitude of fluctuations within the linear noise approximation 9
2.1 Solution of the Lyapunov equation for two-component modules . . 11
2.2 Analysis of noise/susceptibility trading in coherent FFLs 13

3 Power Spectra and Correlations of Fluctuations 14
3.1 Power spectra for two-component networks 15
3.2 Analysis of frequency SNR for different two-component modules 17

4 Regimes of frequency noise filtering 18

5 Optimal detection by two-component modules requires FFL interactions 19

6 General models and numerical simulations 20

7 Model analysis from experimental data 22

1 Response of deterministic two-component networks to constant and oscillatory signals

For a general network of N components, our starting point is the kinetic equations for the evolution of each species

$$\frac{dn_i}{dt} = J_i^+(n_1, n_2, \ldots, n_N) - J_i^-(n_1, n_2, \ldots, n_N), \tag{1.1}$$

where

$n_i \longrightarrow$ macroscopic concentration of the ith molecular species,

$J_i^+ \longrightarrow$ total synthesis rate (production flux),

$J_i^- \longrightarrow$ total degradation rate (relaxation flux),

Following Paulsson [1, 2] we define the elasticities,

$$H_{ij} = -\frac{\bar{n}_j}{\bar{J}_i}\left(\frac{\partial \bar{J}_i^+}{\partial \bar{n}_j} - \frac{\partial \bar{J}_i^-}{\partial \bar{n}_j}\right), \tag{1.2}$$

giving the relative change of the reaction fluxes for component i after a change in the j component. As in main text, we use bars to denote that concentrations and reaction fluxes are evaluated at *equilibrium* ($t \longrightarrow \infty$).

Another quantities of interest are the time scales τ_i defined as [1, 2]

$$\frac{1}{\tau_i} = \frac{\bar{J}_i}{\bar{n}_i}. \tag{1.3}$$

Since $\bar{J}_i^+ = \bar{J}_i^- \equiv \bar{J}_i$, $1/\tau_i$ is usually taken as the degradation rate of the *i*th component assuming linear degradation. We note, however, that this very same definition can be applied to cases of non-linear degradation of a chemical species by a different one, when considering a quasi-steady state approximation as valid [3]. For instance, in the case of hyperbolic consumption of a molecular component n_i by another species n_j in an enzymatic reaction, the relaxation flux can be written as

$$\bar{J}_i^- = \frac{\delta_i \bar{n}_i}{1 + \bar{n}_j/K_j}, \tag{1.4}$$

and one simply defines then an effective decay rate of the form $\frac{1}{\tau_i^{eff}} = \frac{\delta_i}{1+\bar{n}_j/K_j}$.

To quantify the responses of the network we use the *susceptibilities* s_i, defined as the relative change in the concentration of component i at equilibrium, after a change in the input signal,

$$s_i = \frac{\bar{n}_I}{\bar{n}_i} \frac{d\bar{n}_i}{d\bar{n}_I}. \tag{1.5}$$

Hornung and Barkai [4] showed that the susceptibilities can be expressed in terms of the elasticities H_{ij} by differentiating Eq. (1.1) at equilibrium with respect to $\bar{n}_I$. If we consider two isolated interacting molecular species i, j, the susceptibility of species i due to a change in species j is simply

$$s_{ij} = -\frac{H_{ij}}{H_{ii}}. \tag{1.6}$$

We used double index notation here to denote that the susceptibilities s_{ij} give the *local* response of the network element i due to its direct interaction with component j, and we term this as *pairwise* susceptibilities. The susceptibilities s_i defined in Eq. (1.5) give the *global* response of element i due to a change in input signal, and contain the effect of signal propagation through all connected network components. It is straightforward to show following [4] that susceptibilities s_i in a detection network of N components, can be expressed in terms of the pairwise ones solving the algebraic equation

$$\boldsymbol{\mathcal{S}} \cdot \boldsymbol{s} = -\boldsymbol{s_I}, \tag{1.7}$$

where $\mathcal{S}$ is the matrix of pairwise susceptibilities $s_{ij},\ \{i, j = 1, \ldots, N\}$ (with diagonal elements $s_{ii} = -1$) , s the vector of global susceptibilities $s_i,\ \{i = 1, \ldots, N\}$ and $\boldsymbol{s_I}$ the vector of pairwise susceptibilities due to the direct interaction of the input with the network components, $s_{iI},\ \{i = 1, \ldots, N\}$. Solving Eq. (1.7) for $N = 2$ (only sensor and output components) gives Eq. (1) in main text.

If the input is a constant signal, the amplitude of the output response is proportional to the global susceptibility s_O (Fig. S1). If the input is an oscillatory signal, the amplitude of the response will also depend on signal frequency. To see this, consider a single species n with birth/death kinetics whose production rate is periodically forced

$$\frac{dn}{dt} = \alpha \cdot (1 + a_n \sin(\omega t)) - \delta_n n. \tag{1.8}$$

This is a linear differential equation with a time-dependent term which can be readily solved, for instance, by a Laplace transformation. The result is

$$n(t) = \frac{\alpha}{\delta_n} - c_1 \cdot e^{-\delta_n t} + \alpha a_n \frac{\delta_n \sin(\omega t) - \omega \cos(\omega t)}{\omega^2 + \delta_n^2}. \tag{1.9}$$

The second term is a transient, therefore at long times n oscillates around the equilibrium value α/δ_n. The sine and cosine terms can be written in a compact form by using the relation

$$\sin(\omega t + \arg(z)) = \frac{\sin(\omega t)}{|z|} Re(z) + \frac{\cos(\omega t)}{|z|} Im(z), \tag{1.10}$$

from which we get that the oscillating part has the amplitude

$$A(\omega) = \frac{a_n \alpha}{\sqrt{\omega^2 + \delta_n^2}}. \tag{1.11}$$

Therefore, the *squared* amplitude decays as a function of the forcing frequency with a bandwidth $\omega_{BW} = \delta_n$.

If an oscillatory signal is propagated through a network of N components, the output will also oscillate around the stationary mean with the frequency of the input, and an amplitude dependent on this frequency and the network characteristics (time scales, susceptibilities, etc.). Since we are interested in the amplitude of the response around the equilibrium value, we linearize the kinetic equations defining the *relative* deviations from equilibrium as

$$\Delta n_i = \frac{n_i - \bar{n}_i}{\bar{n}_i}, \tag{1.12}$$

and then the amplitude of the relative deviations is given by

$$A_i \equiv \frac{\max[n_i(t)] - \bar{n}_i}{\bar{n}_i}. \tag{1.13}$$

To first order approximation, the relative deviations evolve according to the set of linear equations

$$\frac{\boldsymbol{\Delta n}}{dt} = \boldsymbol{M} \cdot \Delta \boldsymbol{n} + \boldsymbol{q}(t). \tag{1.14}$$

The matrix $\boldsymbol{M}$ is the normalized Jacobian of the kinetic Eqs. (1.1),

$$M_{ij} = \frac{\bar{n}_j}{\bar{n}_i} \left(\frac{\partial \bar{J}_i^+}{\partial \bar{n}_j} - \frac{\partial \bar{J}_i^-}{\partial \bar{n}_j} \right), \tag{1.15}$$

which, using the definition for the elasticities, Eq. (1.2), and for the time scales, Eq. (1.3), can be expressed as [1]

$$M_{ij} \equiv -\frac{H_{ij}}{\tau_i}. \tag{1.16}$$

The vector $\boldsymbol{q}(t)$ contains the explicit time-periodic terms, which can be generally written as

$$q_i(t) = a_i \sin(\omega t + \phi_i). \tag{1.17}$$

Samoilov et al. [5] show that, at long times, the solution of the system of equations (1.14) oscillates with an stationary amplitude given by

$$A(\omega) \stackrel{t\to\infty}{\longrightarrow} |(i\omega \boldsymbol{I} - \boldsymbol{M})^{-1}\boldsymbol{\gamma}|, \tag{1.18}$$

where

$$\gamma_i = a_i e^{i\phi_i}. \tag{1.19}$$

Therefore the relative deviations from equilibrium oscillate with a squared amplitude

$$A^2(\omega) \stackrel{t\to\infty}{\longrightarrow} (i\omega \boldsymbol{I} - \boldsymbol{M})^{-1}\boldsymbol{\gamma}\cdot\boldsymbol{\gamma}^\dagger(-i\omega \boldsymbol{I} - \boldsymbol{M}^T)^{-1}. \tag{1.20}$$

For the specific case of an oscillatory input signal n_I, with dynamics described by Eq. (1.8), acting on two-component network of sensor n_S and output n_O species, the normalized Jacobian matrix reads

$$\boldsymbol{M} = -\begin{pmatrix} \frac{H_{II}}{\tau_I} & 0 & 0 \\ \frac{H_{SI}}{\tau_S} & \frac{H_{SS}}{\tau_S} & \frac{H_{SO}}{\tau_S} \\ \frac{H_{OI}}{\tau_O} & \frac{H_{OS}}{\tau_O} & \frac{H_{OO}}{\tau_O} \end{pmatrix}, \tag{1.21}$$

and the time–periodic vector $\boldsymbol{q}(t)$ is simply

$$\boldsymbol{q}(t) = \begin{pmatrix} \frac{a_I}{\tau_I}\sin(\omega_I t) \\ 0 \\ 0 \end{pmatrix}, \tag{1.22}$$

since we are considering propagation of a single oscillatory signal with amplitude a_I and frequency ω_I, impinging on a network at equilibrium. Then

$$\boldsymbol{\gamma}\cdot\boldsymbol{\gamma}^\dagger = \begin{pmatrix} \frac{a_I^2}{\tau_I^2} & 0 & 0 \\ 0 & 0 & 0 \\ 0 & 0 & 0 \end{pmatrix}. \tag{1.23}$$

We also note that the stability of the equilibrium state of the two-component module is given by the determinant and the trace of its Jacobian matrix. The Jacobian determinant is in this case given by,

$$Jac \equiv \frac{1}{\tau_S\tau_O}(H_{SS}H_{OO} - H_{SO}H_{OS}) = \frac{H_{SS}}{\tau_S}\frac{H_{OO}}{\tau_O}(1 - s_{SO}s_{OS}), \tag{1.24}$$

and the trace by

$$Tr \equiv -\left(\frac{H_{SS}}{\tau_S} + \frac{H_{OO}}{\tau_O}\right). \tag{1.25}$$

If $Jac > 0$ and $Tr < 0$, the network steady state is stable. The self-elasticities are $H_{ii} = 1$ if there is no autoregulation of the i network components, $H_{ii} < 1$ for positive autoregulation and $H_{ii} > 1$ for negative autoregulation. Therefore for the network to be in a stable equilibrium state we should keep $0 < H_{ii} < 1$ for positive autoregulation and $s_{SO}s_{OS} < 1$ in the case of positive feedback.

Using expressions (1.21) and (1.23) in Eq. (1.20) and rearranging terms we arrive to Eqs. (2-3) in the main text.

To check the validity of the linear approximations involved in the response to constant or oscillatory signals, we numerically solved the kinetic equations for a linear genetic cascade (see "Models" section) and obtained the amplitude of the output response for a step input signal of the type

$$\frac{dn_I}{dt} = \alpha_I \cdot (1 + a_I) - \delta_I n_I, \tag{1.26}$$

or for an oscillatory input signal of the form (1.8). Within the linear approximation, the relative change in the output following a step signal of amplitude a_I is given by the $\omega_I \to 0$ limit of Eqs. (2-3) in main text. This limit depends only on signal amplitude and output susceptibility,

$$A^2(0) = \frac{a_I^2 s_O^2}{H_{OO}^2}. \tag{1.27}$$

Thus, for all types of networks and small input perturbations, the relative amplitude A of the output response varies linearly with output susceptibility s_O. This is shown in Fig. S1A for the genetic cascade. At $a_I = 0.01$ (black circles), the response is exactly linear in the whole susceptibility range, while at perturbations higher than 10% of the equilibrium value ($a_I = 0.1$, red circles), it deviates from linearity, but increases monotonically with susceptibility.

For an oscillatory signal the output will oscillate at the same frequency with an amplitude given to linear order by Eqs. (2-3) in main text. In Fig. S1B we show that this approximation reproduces quite accurately the frequency dependent amplitude of a genetic cascade when signal changes up to 50% the equilibrium value ($a_I = 0.5$, blue symbols).

1.1 Frequency detection properties of a linear cascade

As a measure of the frequency detection performance in different modules, we use the range of proper frequency transmission given by the system bandwidth, ω_{BW}.

We employ the standard definition [16]

$$A(\omega_{BW}) \geq \frac{A_{max}}{\sqrt{2}}, \tag{1.28}$$

i.e., the bandwidth is the range of frequencies where the squared amplitude of the response is above one half of its maximum value. For systems behaving as low-pass filters, such as the linear cascade, A_{max} is given by the amplitude at $\omega_I = 0$. Therefore ω_{BW} is given by the solution of the equation

$$A^2(\omega_{BW}) = \frac{A^2(0)}{2}. \tag{1.29}$$

A look at Eq. (4) in main text shows that, for fixed time scales, the bandwidth is independent of the output susceptibility s_O. How does it depend on the time scales of the cascade components? First, one sees that when all degradation rates δ_i are similar, the larger the degradation rate (faster time scales) the larger the bandwidth, and viceversa (compare red curve with $\delta_I = \delta_S = \delta_O = 2$ and black curve, $\delta_I = \delta_S = \delta_O = 1$, in Fig. S2A). Moreover, being the product of three low-pass filters, the bandwidth is limited by the slowest time scale of the system. This is illustrated in Fig. S2A (blue curve), with the response of a linear cascade where the module components, sensor and output species, are fast ($\delta_S = \delta_O = 2$) but the input signal has a lower degradation rate ($\delta_I = 1$) limiting the range of transmitted frequencies. In this case, the response is exactly the same if the slowest time scale is either in the sensor or the output species. Another feature of linear cascades is that adding successive layers slows down output response [6]. A single component with periodically forced production rate, Eq. (1.8), has a bandwidth given by its degradation rate, Eq. (1.11) and dot-dashed line/shaded region in Fig. S2B. If this single oscillatory component is taken as an input acting on a second element, the output response is given by the solution of Eq. (1.20) with

$$\boldsymbol{M} = -\begin{pmatrix} \frac{H_{II}}{\tau_I} & 0 \\ \frac{H_{OI}}{\tau_O} & \frac{H_{OO}}{\tau_O} \end{pmatrix}, \tag{1.30}$$

which yields

$$A^2(\omega_I) = \frac{a_I^2}{\tau_I^2} \frac{s_{OI}^2 H_{OO}^2/\tau_O^2}{\left(\omega_I^2 + \frac{H_{II}^2}{\tau_I^2}\right)\left(\omega_I^2 + \frac{H_{OO}^2}{\tau_O^2}\right)}. \tag{1.31}$$

Even for the same time scales, $\delta_I = \delta_O = 1$, the second filter reduces the bandwidth, dashed line in Fig. S2B. The bandwidth is further decreased by adding an intermediate species, and thus forming a three-layered cascade (solid line in Fig. S2B and Eq. (4) in main text).

1.2 Simultaneous determination of bandwidth and susceptibility for two-component networks

In order to compare amplitude/frequency detection features of two-component network motifs in a consistent way, we fix the sign and susceptibility of the input/sensor interaction, which we denote as s^*_{SI} ($s^*_{SI} = 2$ for Figures in main text). Each two-component module is then characterized by the sign and ranges of its remaining interactions, given as pairwise susceptibilities s_{ij}, and by the time scales of each component, given as the inverse of the degradation rates δ_i. Throughout the paper we take similar time scales for each node in the network, including the signal, and fix $\delta_i = 1$. We also allow pairwise susceptibilities to vary in the range $s_{ij} \in [smin, smax]$. We take $smin = 0$ and $smax = 5$ in main text.

Next we find, for the simplest two-component networks analyzed here (positive and negative feedbacks, positive and negative autoregulations, and coherent/incoherent feed-forward loops) which is the relevant interaction parameter determining the bandwidth of the oscillatory response. For the feedback module (both positive and negative), the difference with the linear cascade comes only through the extra term in the denominator, Eqs. (3) in main text. Then the difference in bandwidth, for fixed time scales, depends only upon the product of susceptibilities $s_{OS}s_{SO}$. To compare positive and negative feedbacks on the same footing we take the absolute value of this product and denote it as *feedback strength* (FS). To calculate the bandwidth of the oscillatory response as a function of FS, we generate a random uniform distribution of pairwise susceptibilities s_{OS} and s_{SO} in the interval $[smin, smax]$ [4] (providing all possible FS values), calculate the theoretical response amplitude using Eqs. (2)-(3) in main text and obtain the bandwidth. Plotting the bandwidth as a function of FS we obtain the black curves in Fig. 2 in main text. Next, we determine the output susceptibility range compatible with each FS value. This is easily obtained from Eq. (1) in main text. For instance, for negative feedback the output susceptibility is

$$s_O = \frac{s_{OS}s^*_{SI}}{1 + FS}. \tag{1.32}$$

Therefore, for each FS value the maximum possible susceptibility s_O corresponds to the case $s_{OS} = smax$ giving the upper red curve in Fig. 2C. The minimum value of s_O, on the other hand, is obtained by the minimum value of sensor/output susceptibility s_{OS} compatible with a given FS, which is $s_{OS} = FS/smax$ (if $smin = 0$), and generates the lower red curve in Fig. 2C. All possible combinations of s_{OS} and s_{SO} values will give output susceptibilities s_O lying between these curves (grey shaded region in Figures 2C,D).

For modules with autoregulation in one of the components, let's say the output

element, $H_{OO} \neq 1$, we define the *autoregulation strength* (ARS) as

$$ARS \equiv |H_{OO} - 1|. \tag{1.33}$$

The amplitude/frequency detection features for negative(positive) autoregulation as a function of ARS are similar to those for negative(positive) feedback and we plot them in Fig. S3: increasing ARS in negative autoregulation improves bandwidth but decreases amplitude detection, while the opposite behavior is observed for positive autoregulation.

For modules with feed-forward connections between input and output elements, $s_{OI} \neq 0$, the bandwidth is determined by the relative contribution of the second term in the numerator of Eq. (7) in main text, acting as a high-pass filter. Thus we define the *relative strength* (RS$\equiv$ $|s_{OI}/s_O|$) as the relevant interaction parameter for frequency detection in feed-forward loops. It is clear that the high-pass filter term dominates when $s_{OI}^2 \gg s_O^2$, which is only possible for incoherent FFLs, where $sign(s_{SI} \cdot s_{OS}) \neq sign(s_{OI})$. This regime of high-pass filtering of an oscillatory signal effectively takes place for RS> 3.3 (Fig. 3 in main text), meaning that low frequency oscillations are transmitted with an amplitude smaller than $A_{max}/\sqrt{2}$ (strictly speaking, circuits in this regime are *band-pass* filters, since there is always a cutoff at very high frequencies due to the intrinsic time scales of the components, but we use the denomination *high-pass* in order to stress the difference with the low-pass regime at fixed time scales). The high-pass behavior of the incoherent FFL (illustrated with trajectories in Fig. 3F in main text) is shown in Fig. S4 for the whole range of input frequencies (grey symbols) at RS=6. In order to compare with the negative feedback case at the same susceptibility and similar frequency detection regime (FS=6), we also plot the negative feedback oscillatory response with black circles.

The relevant interaction parameters determining frequency detection, together with the corresponding susceptibility ranges for each of these parameter values, are provided in Table S1.

2 Amplitude of fluctuations within the linear noise approximation

For small number of molecules, as it is usually the case in signaling reactions inside cells, the proper mathematical framework to describe the network dynamics is the master equation for the evolution of probabilities of the number of molecular species [7]. The kinetic equations (1.1) describe only the evolution of average number of molecules [12] (related to the macroscopic concentrations n_i by the "system size" or volume factor V as $\langle n_i \rangle = V n_i$) . The amplitude of the fluctua-

tions around this average numbers *at equilibrium*, assuming small fluctuations, is usually obtained following two alternative routes

1) Starting from the master equation, there can be derived *exact* equations for the time evolution of first and second moments of molecule numbers [8, 9]. Linearizing around the equilibrium state, one obtains a Lyapunov algebraic equation for the covariance of the fluctuations around steady state [9, 10]. The linear approximation should be valid in principle whenever the equilibrium point is far from a bifurcation, and the size of the fluctuation is not too large. Numerical simulations show that for steady states far from bifurcations, this is a good approximation even for large fluctuations. For an analysis of fluctuations using the Lyapunov equation near critical points see [11]. With the same notation used in Section 1 for reaction fluxes and elasticities, the Lyapunov equation can be expressed as [1, 2]

$$\boldsymbol{M} \cdot \boldsymbol{\sigma} + \boldsymbol{\sigma} \cdot \boldsymbol{M^T} + \boldsymbol{D} = \boldsymbol{0}, \tag{2.34}$$

where $\boldsymbol{M}$ is the normalized Jacobian matrix, whose elements are defined as in Eq. (1.16); $\boldsymbol{\sigma}$ is the matrix of normalized covariances, $\sigma_{ij} = \langle (n_i - \bar{n}_j)(n_j - \bar{n}_j) \rangle / \bar{n}_i \bar{n}_j$ (note that σ_{ii} is the squared coefficient of variation for fluctuations of the i component), and $\boldsymbol{D}$ the diffusion matrix whose elements depend on the reaction fluxes, system size and stoichiometric coefficients [1, 2]. To solve the algebraic equation (2.34) we assume that each molecular reaction affects a single species (which is not always the case, but it holds for the genetic networks studied here) and thus $D_{ij} = 0, \, \forall j \neq i$. For an analysis of cases where the diffusion matrix is not diagonal but can be transformed to diagonal and the Lyapunov equation solved, see Ref. [9]. Moreover, if each reaction adds or removes a single molecule, the diagonal diffusion elements, which give the individual noise strengths D_i, take the simple form [1]

$$D_i \equiv D_{ii} = \frac{2}{V \bar{n}_i} \frac{1}{\tau_i}, \tag{2.35}$$

where V is the system volume. In practice we take a volume factor for each molecular species (V_i) as an effective parameter to change the noise strengths. Although we use Eq. (2.35) for the systems investigated here, all analytical results are expressed in terms of general noise strengths D_i. For instance, if species i is produced as a burst of b molecules condensed in a single reaction, while decaying linearly with a time constant τ_i (a situation commonly considered for mRNA transcription in prokaryotic gene expression [8]),

$$\phi \xrightarrow{k_i} b \, x_i, \qquad x_i \xrightarrow{1/\tau_i} \phi, \tag{2.36}$$

its noise strength takes the form

$$D_i = \frac{(1+b)}{\tau_i V \bar{n}_i}. \tag{2.37}$$

2) A second derivation starts again from the master equation, and performs a system size expansion [12] which can be used to derive Langevin equations for the dynamical variables. The approximations involved in this procedure are discussed in Ref. [7]. Linearizing around the equilibrium states, one gets again a system of linear Langevin equations for the time evolution of the fluctuations. These define a multivariate Ornstein-Uhlenbeck process which can be solved by standard methods (Gardiner (1985) [13]), yielding again the Lyapunov equation (2.34).

Both approaches are equivalent and thus no additional approximations are involved. The advantage of using the Langevin formalism is that, as we see below, the power spectrum of the fluctuations can be readily obtained from the same matrix definitions as above.

2.1 Solution of the Lyapunov equation for two-component modules

It is instructive to consider first the solution of the Lyapunov equation (2.34) for a general system of two species, n_1 and n_2. The Jacobian and diffusion matrices read

$$\boldsymbol{M} = -\begin{pmatrix} \frac{H_{11}}{\tau_1} & \frac{H_{12}}{\tau_2} \\ \frac{H_{21}}{\tau_1} & \frac{H_{22}}{\tau_2} \end{pmatrix}, \ \boldsymbol{D} = \begin{pmatrix} D_1 & 0 \\ 0 & D_2 \end{pmatrix}. \tag{2.38}$$

Species 1 may stand for mRNA and 2 for protein, for instance, and then we can study different forms of autoregulation. If they denote different proteins, one can treat all kind of feedback interactions between both species, whenever the condensed two-variable description is a proper approximation. The aim is to express the noise covariances in terms of the same deterministic quantities (susceptibilities, time scales and elasticities) used for the description of the amplitude and frequency response in Section 1. The result for the autocovariances is

$$\sigma_1 = \sigma_1^{int}\left(1 + s_2\frac{H_{12}/\tau_1}{T_{av}}\right) + \sigma_2^{int} s_1^2 \frac{H_{11}/\tau_1}{T_{av}}, \tag{2.39}$$

$$\sigma_2 = \sigma_2^{int}\left(1 + s_1\frac{H_{21}/\tau_2}{T_{av}}\right) + \sigma_1^{int} s_2^2 \frac{H_{22}/\tau_2}{T_{av}}, \tag{2.40}$$

where

$$T_{av} \equiv -Tr(\boldsymbol{M}) = \frac{H_{11}}{\tau_1} + \frac{H_{22}}{\tau_2}, \tag{2.41}$$

as in Eq. (1.25),

$$\begin{aligned} s_1 &= -\frac{H_{12}}{H_{11}}, \\ s_2 &= -\frac{H_{21}}{H_{22}}, \end{aligned} \tag{2.42}$$

are the susceptibilities of species 1 and 2 respectively (in this case global and pairwise susceptibilities are the same), and the intrinsic noise prefactors are given by

$$\sigma_i^{int} = \frac{D_i \tau_i}{2} \frac{1}{H_{ii} + s_j H_{ij}}. \tag{2.43}$$

As discussed by Paulsson [1,2] T_{av} can be interpreted as the total rate for returning to equilibrium (time scale for relaxation) after intrinsic fluctuations. For the case $H_{12} = 0$, and with the noise strengths D_i given by Eq. (2.35), the expression for the autocovariances, Eqs. (2.39)-(2.40), recover that of Ref. [1]. For a general H_{12} it has the same form, except that intrinsic noise amplitudes are corrected by the susceptibilities. In any case, the coefficient of variation of fluctuations for each component n_i can be separated into an intrinsic contribution due to its own fluctuations corrected by terms proportional to $s_j H_{ij}$ (expressing how the action of n_i on the other component feeds back on itself), plus a forced or extrinsic contribution arising from fluctuations in the other component –weighted by the static susceptibility and time averaging.

Next we solve the case studied here, that of a signal acting on a two-component module, with the Jacobian matrix given by Eq. (1.21). The only restriction is that signal dynamics is not affected by any of the module components. For a discussion on the effect of correlations among signal and downstream components see Ref. [14]. Proceeding as previously, solving the Lyapunov equation (2.34) and rearranging the results in terms of susceptibilities and time scales we arrive at the following expression for the autocovariance of the output fluctuations:

$$\begin{aligned} \sigma_O &= \sigma_O^{int}\left(1 + s_{SO}\frac{H_{OS}/\tau_O}{T_{av}}\right) \\ &+ \sigma_S^{int} s_{OS}^2 \frac{H_{OO}/\tau_O}{T_{av}} \\ &+ \sigma_I^{int}\left(s_O^2 \frac{T_{av}^{tot}}{T_{av}} \frac{Jac}{Jac + \frac{H_{II}}{\tau_I} T_{av}^{tot}} + s_{OI}^2 \frac{H_{OO}/\tau_O}{T_{av}} \frac{\frac{H_{II}}{\tau_I}\frac{H_{OO}}{\tau_O}}{Jac + \frac{H_{II}}{\tau_I} T_{av}^{tot}}\right), \end{aligned} \tag{2.44}$$

where Jac is the two–component Jacobian determinant given by Eq. (1.21), T_{av}^{tot} the total relaxation time for intrinsic fluctuations,

$$T_{av}^{tot} \equiv H_{II}/\tau_I + H_{SS}/\tau_S + H_{OO}/\tau_O, \tag{2.45}$$

and the intrinsic noises σ_i^{int} are again given by Eq. (2.43).

The first two terms in the previous Eq. (2.44) are identical to the two-species system discussed above, Eq. (2.40), and represent again the effect of intrinsic output fluctuations (first term) corrected by possible feedback of the output on the sensor element, plus the fluctuations induced in the output by the sensor species itself (second term). The third term represents the effect of signal noise propagated through the two-component network. It contains two contributions: one weighted by the total susceptibility s_O^2, which takes into account signal noise propagated to the output through the sensor element, and other (present in FFL motifs) depending on the direct input/output interaction s_{OI}^2. The case with $\sigma_S^{int} = \sigma_O^{int} = 0$ (only signal noise) has been analyzed by Hornung and Barkai [4], where they have shown that for the simple circuits studied here, positive feedback (or autoregulation) is the only architecture giving noise reduction with respect to the linear cascade at similar susceptibility values. The importance of taking into account all possible noise contributions is discussed in the next section, taking the coherent FFL as an example.

In Fig. S5A, the theoretical prediction for the output noise variance Eq. (2.44), expressed as a coefficient of variation, is numerically validated with Gillespie simulations [15] for three different networks: a linear cascade (red), a coherent FFL (blue) and an incoherent FFL (green). The noise strength of sensor and output species is fixed through their "system size" parameters $V_S = V_O = 1$, and signal noise is varied monotonically increasing V_I. We note that for the same susceptibility the coherent FFL (blue) can be tuned to decrease total output noise with respect to the linear cascade.

2.2 Analysis of noise/susceptibility trading in coherent FFLs

From the results of Fig. 5B in main text and the previous Fig. S5A we conclude that coherent FFLs usually have better SNR in amplitude than linear cascades. In order to see how this is possible, we compare the total output noise for both networks at the same total susceptibility, $s_O^{\text{lc}} = s_O^{\text{cffl}} \equiv s_O$ (recall that input/sensor interaction s_{SI} is also fixed). To simplify the analysis, we assume equal time scales and noise strengths in all components: $\tau_I = \tau_S = \tau_O \equiv \tau$, $D_I = D_S = D_O \equiv D$. Then substituting in Eq. (2.44) the output noise variance for the linear cascade can be written as

$$\sigma_O^{\text{lc}} = \frac{D\tau}{2}\left(1 + \frac{s_O^2}{2s_{SI}^2} + \frac{3}{8}s_O^2\right), \tag{2.46}$$

where we have used the relation $s_{OS} = s_O/s_{SI}$ for the linear cascade. The first, second and third terms are the contributions of intrinsic, sensor propagated and signal propagated fluctuations on output in "units" of noise amplitudes $D\tau/2$. Proceeding in the same way, we have for the coherent FFL

$$\sigma_O^{\text{fflc}} = \frac{D\tau}{2}\left[1 + \frac{(s_O - s_{OI})^2}{2s_{SI}^2} + \frac{1}{8}(3s_O^2 + s_{OI}^2)\right], \tag{2.47}$$

where we have used $s_{OS} = (s_O - s_{OI})/s_{SI}$ for the sensor/output susceptibility in coherent FFLs. Then we see that the extra direct interaction s_{OI} adds noise to the term accounting for signal noise propagation(third term in brackets), but *reduces* noise propagated through the sensor species (second term). We can calculate the conditions for which the effective noise reduction in the sensor species is larger than signal noise amplification. Expressing everything in terms of the relative strength (RS) defined above for feed-forward loops, we find that

$$RS \leq \frac{8}{4 + s_{SI}^2}, \tag{2.48}$$

for total noise reduction, which depends only on the direct input/sensor interaction, and not on total susceptibility. Thus, for $s_{SI} \leq 2$ we have noise levels in the coherent FFL lower than those of a linear cascade at equivalent signal sensitivity (recall that $RS \leq 1$ for the coherent FFL, and that this analysis holds for equal noise strengths and time scales). This is illustrated in Fig. S5C, where we plot output coefficient of variation as a function of output susceptibility at $s_{SI} = 2$ for the linear cascade (red) and the coherent FFL (blue). The rest of the interactions are varied sampling the interval $s_{ij} \in [0, 5]$ uniformly, as in Fig. 5 in main text.

3 Power Spectra and Correlations of Fluctuations

To analyze fluctuations in the frequency domain, it is useful to start from the Langevin approximation for the stochastic dynamics [7,12,17]. Linearizing around steady sate, one gets linear evolution equations for the fluctuations around equilibria. The dynamics for the *normalized* fluctuations $\eta_i = \frac{n_i - \bar{n}_i}{\bar{n}_i}$ is given by

$$\frac{d\eta_i}{dt} = \sum_{j=1}^{N} M_{ij}\eta_j + \zeta_i, \tag{3.49}$$

where M_{ij} are the normalized Jacobian matrix elements defined by Eqs. (1.15)-(1.16), and ζ_i are Gaussian white noises obeying the fluctuation dissipation theorem

$$\langle \zeta_i(t)\zeta_j(0)\rangle = D_{ij}(\boldsymbol{n})\delta(t), \tag{3.50}$$

where the diffusion matrix $\boldsymbol{D}$ is the same appearing in Eq. (2.34). Eqs. (3.49)-(3.50) define a multivariate Ornstein-Uhlenbeck process whose solution is well known [10,13]. The power spectrum matrix in stationary state, $P_{\eta_{ij}}(\omega) = \langle \hat{\eta}_i(\omega)\hat{\eta}_j(\omega)\rangle$ follows as [13]

$$P_{\boldsymbol{\eta}}(\omega) = \frac{1}{2\pi}\left(\boldsymbol{M} + \boldsymbol{I}i\omega\right)^{-1}\boldsymbol{D}\left(\boldsymbol{M}^T - \boldsymbol{I}i\omega\right)^{-1}. \tag{3.51}$$

We note that the correlations $C_{ij} = \langle \eta_i(t)\eta_j(0)\rangle$ can be also obtained directly from the Langevin formulation using the regression theorem [13, 17]

$$\frac{d\boldsymbol{C}}{dt} = \boldsymbol{C}\cdot\boldsymbol{M}^T, \tag{3.52}$$

that after a Laplace transformation leads to the set of linear algebraic equations

$$s\tilde{\boldsymbol{C}}(s) = \tilde{\boldsymbol{C}}(s)\cdot\boldsymbol{M}^T + \boldsymbol{\sigma}, \tag{3.53}$$

with $\boldsymbol{\sigma}$ being the variance matrix. The power spectra can also be calculated from the Laplace transform of the correlations as

$$P_{\eta_{ii}}(\omega) = \tilde{C}_{ii}(i\omega) + \tilde{C}_{ii}(-i\omega). \tag{3.54}$$

3.1 Power spectra for two-component networks

Proceeding as for the noise variance, we solve first Eq. (3.51) for the simpler case of two interacting molecular species n_1 and n_2 (mRNA/protein, protein 1/protein 2, etc.) with Jacobian and diffusion matrices given by Eq. (2.38). After some algebra we rearrange terms as

$$P_{\eta_{11}}(\omega) = D_{11}\frac{\omega^2 + \frac{H_{22}^2}{\tau_2^2}}{\Delta(\omega)} + D_{22}s_1^2\frac{H_{11}^2/\tau_1^2}{\Delta(\omega)}, \tag{3.55}$$

$$P_{\eta_{22}}(\omega) = D_{22}\frac{\omega^2 + \frac{H_{11}^2}{\tau_1^2}}{\Delta(\omega)} + D_{11}s_2^2\frac{H_{22}^2/\tau_2^2}{\Delta(\omega)}, \tag{3.56}$$

where the denominator is

$$\Delta(\omega) \equiv \left(\omega^2 + \frac{H_{11}^2}{\tau_1^2}\right)\left(\omega^2 + \frac{H_{22}^2}{\tau_2^2}\right) + 2\frac{H_{12}H_{21}}{\tau_1\tau_2}\left(\omega^2 + \alpha\right), \tag{3.57}$$

and

$$\alpha \equiv \left(\frac{s_{12}s_{21}}{2} - 1\right)\frac{H_{11}}{\tau_1}\frac{H_{22}}{\tau_2}. \tag{3.58}$$

Similarly to the case of noise variance, the power spectrum for species n_i can be decomposed into an intrinsic contribution whose magnitude at zero frequency is proportional to its noise strength D_i, and an extrinsic contribution depending on the other species' fluctuations and susceptibility. Consider the case that n_1 acts directly upon n_2 with no feedback ($H_{12} = 0$)

$$P_{\eta_{11}}(\omega) = \frac{D_{11}}{\left(\omega^2 + \frac{H_{11}^2}{\tau_1^2}\right)}, \tag{3.59}$$

$$P_{\eta_{22}}(\omega) = \frac{D_{22}}{\left(\omega^2 + \frac{H_{22}^2}{\tau_2^2}\right)} + D_{11}s_2^2 \frac{H_{22}^2/\tau_2^2}{\left(\omega^2 + \frac{H_{11}^2}{\tau_1^2}\right)\left(\omega^2 + \frac{H_{22}^2}{\tau_2^2}\right)}. \tag{3.60}$$

Then one sees that n_1 (input) has the spectrum of a pure Poisson or birth/death process, which is a low-pass filter with bandwidth $\omega_{BW} = H_{11}/\tau_1$, whereas the n_2 spectrum (output) has an intrinsic part given by its own Poissonian filter with bandwidth H_{22}/τ_2, plus an extrinsic contribution which is the product of the input and output filters scaled by the static susceptibility [14, 18].

Solving Eq. (3.51) for our input/sensor/output network with Jacobian matrix Eq. (1.21) is equally straightforward and gives Eq. (11) in main text for the output component. We recall that this gives the spectrum of stationary fluctuations after the output element reached steady state (for instance, following a change in signal amplitude). For an oscillatory signal with noise, the output spectrum $P(\omega)$ can be decomposed into an oscillatory part and a noise background [19],

$$P(\omega) = P_{fluc.}(\omega) + P_{osc.}(\omega), \tag{3.61}$$

where the background term $P_{fluc.}(\omega)$ is the power spectrum of the output fluctuations around the mean, Eq. (11) in main text, and the oscillatory component is given by

$$P_{osc.}(\omega) = \frac{A^2(\omega_I)}{2\pi}\left[\delta(\omega - \omega_I) + \delta(\omega + \omega_I)\right]. \tag{3.62}$$

Here $A(\omega_I)$ is the amplitude of the output oscillations, given to linear approximation by Eq. (2) in main text. The signal-to-noise ratio (SNR) can be calculated as [19]

$$SNR_{freq}(\omega_I) = \frac{2\left[\lim_{\delta\omega\to 0}\int_{\omega_I-\delta\omega}^{\omega_I+\delta\omega} P_{osc.}(\omega)d\omega\right]}{P_{fluc.}(\omega_I)} = \frac{A^2(\omega_I)}{\pi P_{fluc.}(\omega_I)}. \tag{3.63}$$

To check the validity of the above expressions we show the numerical output power spectra of three different genetic circuits in Fig. S5B. The theoretical background spectra given by Eq. (11) in main text are plotted in red (linear cascade), blue (coherent FFL) and green (incoherent FFL). The height of the peaks at the

oscillatory input frequency $\omega_I = 1$, marked with crosses, are proportional to the amplitudes $A^2(\omega_I)$. This is shown in the inset of Fig. S5B, where we plot the theoretical $A^2(\omega_I)$ given by Eq. (2) in main text for the linear cascade (red line) and incoherent FFL (green line) and the numerical peak heights of those modules at different input frequencies (black circles). We note that the numerical power spectra are divided by the bin size $\Delta\omega$ (the frequency resolution) to be independent of the total sampling time, and thus are power spectral densities (power per unit frequency). While the background term $P_{fluc.}(\omega)$ is a power spectral density, the oscillatory term in Eq. (3.62) is the product of a δ function (with units of inverse frequency) and the total power under it, therefore in order to compare with the numerical power spectra the theoretical oscillatory power must be divided by the resolution $\Delta\omega$ [20].

3.2 Analysis of frequency SNR for different two-component modules

Similar to the analysis of noise/susceptibility features for the coherent FFL above, we simplify parameters not depending on circuit architecture as much as possible, and assume again equal time scales τ and noise strengths D for all network elements. Since we are comparing SNR of different circuits at input frequencies where frequency transmission is maximum [$A(\omega_I) = A_{max}$], this usually happens at $\omega_I \sim 0$ (except for the incoherent FFL which can behave as a high-pass filter for oscillatory signals). Substituting the theoretical expressions for $P_{fluc.}(\omega_I)$ and $A^2(\omega_I)$ in Eq. (3.63) and taking the limit $\omega_I \to 0$ we have the following expression for the SNR

$$SNR_{freq}(0) = \frac{a_I^2\tau^2}{D}\frac{s_O^2 Jac^2}{1 + s_{OS}^2 + s_O^2 Jac^2\tau^4}, \qquad (3.64)$$

where Jac is given by Eq. (1.24). Then, for linear cascades and FFLs with no autoregulation, $Jac^2 = \tau^4$ and the SNR reads

$$SNR_{freq}^{lc,ffl}(0) = \frac{a_I^2}{D\tau^2}\frac{s_O^2}{1 + s_{OS}^2 + s_O^2}. \qquad (3.65)$$

As in the analysis of noise amplitude above, we recall that $s_{OS} = s_O/s_{SI}$ for the linear cascade, but $s_{OS} = (s_O - s_{OI})/s_{SI}$ for the FFL. Therefore, for the same output susceptibility s_O, giving the same oscillatory amplitude at $\omega_I \to 0$, Eq. (1.27), the coherent FFL ($s_{OI} > 0$) will have a frequency SNR larger than the linear cascade. The reason, as in the previous analysis, is again the reduction in the contribution to background power spectrum of the noise propagated through sensor component. This is shown in Fig. S5D where $P_{fluc.}(\omega_I)$ is plotted versus $A^2(\omega_I)$ at maximum transmission for the same sampling used in Fig. S5B.

For the feedback interactions the situation is different, since $Jac^2 = \tau^4(1 - FS)^2$ [$Jac^2 = \tau^4(1 + FS)^2$] for positive(negative) feedback. Substituting in Eq. (3.64) we see that positive feedback always decreases SNR_{freq} with respect to a linear cascade with the same oscillatory amplitude, while negative feedback has the opposite effect. Thus the frequency SNR behaves differently than the amplitude SNR. While amplitude SNR improves in positive feedback due to time averaging [4], frequency SNR deteriorates due to loss of stability of the steady state (in the limit $FS \to 1$ the steady state becomes unstable and $SNR_{freq} \to 0$). Another observation for feedback regulation is that $s_O^2 Jac^2 = s_{OS}s_{SI}$ which is the susceptibility of a linear cascade; therefore the maximum SNR_{freq} at any feedback strength can not be larger than the maximum SNR_{freq} of the linear cascade with the same interaction strengths (as it is shown in Fig. 4C–D in main text).

4 Regimes of frequency noise filtering

Two terms potentially giving high–pass filter behavior appear in the fluctuation power spectrum, Eq. (11) in main text. One of them is induced by the intrinsic output fluctuations [first term in Eq. (11)], and may be at work whenever the second term in the denominator $\Delta(\omega)$ is different from zero. This happens if there is a feedback interaction between the output and the sensor species. Intuitively, one could expect that a negative feedback, which accelerates dynamics [21] will be able to accelerate also the intrinsic fluctuations. This has been demonstrated theoretically [22] and experimentally [23] by Simpson and coworkers. They showed that negative feedback in simple gene autoregulation or in a two-gene system produced a shift of the power spectral density to higher frequencies. This result can be also deduced from Eq. (11) by explicitly calculating the bandwidth of the module noise power spectrum. Expressing the denominator $\Delta(\omega)$ in the alternative form

$$\Delta(\omega) = Jac^2 + \Gamma\omega^2 + \omega^4 \tag{4.66}$$

where Jac is the Jacobian determinant defined in Eq. (1.24) and Γ is defined as

$$\Gamma = \frac{H_{SS}^2}{\tau_S^2} + \frac{H_{OO}^2}{\tau_O^2} + 2\frac{H_{SO}H_{OS}}{\tau_S\tau_O} \tag{4.67}$$

the bandwidth frequency of the module fluctuations is given by

$$\omega_{BW}^{mod.fluc.} = \frac{1}{H_{SS}/\tau_S}\sqrt{2Jac^2 - \Gamma\frac{H_{SS}^2}{\tau_S^2} + \sqrt{4\frac{H_{SS}^4}{\tau_S^4}Jac^2 + \left(\frac{H_{SS}^2}{\tau_S^2}\Gamma - 2Jac^2\right)^2}} \tag{4.68}$$

In a negative feedback, the elasticities H_{SO} and H_{OS} have opposite signs. Therefore the Jacobian determimant (1.24) increases and the parameter Γ decreases, which from Eq. (4.68) it is seen to increase the frequency of the fluctuations.

To find the regimes where the time scale of fluctuations can be well separated in frequency detection, we calculate the overlap between the bandwidth of $A^2(\omega_I)$ and the bandwidth of the fluctuation spectrum, $P_{fluc}(\omega_I)$. If this overlap is smaller than the oscillatory bandwidth, there is a range of output frequency response free of fluctuations. To quantify this filtering capacity, we define the *filter range* (FR) by

$$FR = \frac{\omega_{BW}^{osc} - ovlp}{\omega_{BW}^{osc}} sign\left(\omega_{max}^{osc} - \omega_{max}^{fluc}\right), \tag{4.69}$$

where ω_{BW}^{osc} is the oscillations bandwidth, $ovlp$ is the overlap between oscillations and fluctuations bandwidth, ω_{max}^{osc} is the maximum oscillation frequency transmitted, and ω_{max}^{fluc} the maximum fluctuation frequency transmitted. Therefore, if $FR = 0$, there is no filter range, since $ovlp = \omega_{BW}^{osc}$. For $0 < |FR| < 1$, there is a range in oscillation bandwidth free of noise. If $|FR| > 1$ the system works as a perfect filter, completely separating the time scale of transmitted oscillations and fluctuations for the whole circuit bandwidth. The sign of FR indicates if the system filters fast fluctuations ($FR < 0$, since $\omega_{max}^{fluc} > \omega_{max}^{osc}$) or slow fluctuations ($FR > 0$).

As suggested by theory, we found only two type of circuits which could filter out fluctuations by frequency content. In Fig. S6A, we show how the FR of negative feedback circuits changes as a function of FS. We see that for $FS > 5$, ($FR < -1$, shaded region in Fig. S6A) a negative feedback module can behave as a perfect filter completely separating fluctuations from transmitted oscillatory frequencies (since fluctuations are shifted to frequencies larger than oscillatory response bandwidth, Fig. 6A in main text). On the other hand, incoherent FFLs may act as partial noise filters ($0 < FR < 1$, Fig. S6B) when input oscillations are at high frequencies, since in this case the oscillatory response is large and fluctuations are always slower (Fig. 6B in main text).

5 Optimal detection by two-component modules requires FFL interactions

In the main text we have analyzed the simplest two–component detection motifs (feedbacks, autoregulations and FFLs). Mixed modules, made of a combination of two or more 'pure' motifs, may be able to improve simultaneous amplitude/frequency signal detection exploiting the particular advantages of each

simple architecture. To explore this possibility, we have generated a random sample (10^5 elements) of all possible two–component modules with a uniform distribution of interaction strengths in the range $s_{ij} \in [0, 5]$ as in Figures 2-5 in main text. Since we also fixed the direct input-sensor interaction ($s_{SI} = 2$, we have two possibilities for the sensor-output interaction (positive or negative) plus three possibilities (positive, negative or zero) for the remaining four interactions (feed–forward, feedback and two autoregulations), giving $2 \times 3^4 = 162$ detection circuits. We calculated output susceptibility, bandwidth, amplitude SNR and frequency SNR for each motif in the sample. We first selected those sampling elements giving both susceptibility and bandwidth larger than the linear cascade limit ($s_O > 10, \omega_{BW} > \omega_{BW}^{LC} = 0.51$) and identified the specific circuits. For each of those modules we calculated the frequency of appearance in the selected (s_O, ω_{BW}) region, divided by the frequency of appearance in the whole (s_O, ω_{BW}) space (Relative frequency, shown as histograms in Figure S7A). In this way, relative frequencies larger than unity indicate that the specific motif is not only capable of simultaneous amplitude and frequency optimal detection, but also that optimal detection is robust, since for random susceptibilities there is more probability of appearance in the optimal region than in the rest of the space. We found, Figure S7A, that most of the circuits in the selected regime are pure coherent FFLs. The only mixed modules which appear with a significative frequency are incoherent FFLs with a combination of feedbak or autoregulations of different signs, but in a much smaller proportion than coherent FFLs. We have used the same procedure to obtain the relative frequency of motifs with optimal signal detection with noise, selecting those circuits with both amplitude and frequency SNRs larger than the maximum value allowed in a linear cascade configuration (Figure S7B). Again, motifs involving coherent FFLs are necessary for optimal detection, although mixed FFLs with positive feedback or autoregulation appear with similar relative frequencies than pure coherent FFLs. This is in contrast with the noiseless situation, Figure S7A.

6 General models and numerical simulations

The three component networks (input, sensor and output) numerically simulated in Figures 2 and 3 of the main text follow the same general system of differential equations

$$\begin{aligned} \frac{dn_I}{dt} &= \alpha_I(1 + a_I \sin \omega_I t) - \delta_I n_I, \\ \frac{dn_S}{dt} &= \alpha_S F_S - \delta_S n_S, \end{aligned}$$

$$\frac{dn_O}{dt} = \alpha_O F_O - \delta_O n_O, \quad (6.70)$$

($\omega_I = 0$ for constant input signals) with linear degradation rates δ_X. The production terms F_S and F_O are given in terms of Hill functions f_{act}, f_{rep} for activation and repression of the form

$$f_{act}(n_X, k_X) = \frac{(n_X/k_X)^n}{1 + (n_X/k_X)^n},$$
$$f_{rep}(n_X, k_X) = \frac{1}{1 + (n_X/k_X)^n}, \quad (6.71)$$

where k_X is the corresponding activation/repression threshold and n the Hill coefficient [24].

The expressions for sensor and output production terms in the two-component modules simulated are listed in Table 1. For positive feedback and coherent FFLs we use a function with OR logic for production of sensor and output species respectively. The reason for this choice is that the linear cascade architecture is recovered in the limit of large activation thresholds for the additional interactions ($f_{act}(\mathrm{O}, k_O \to \infty)$, $f_{act}(\mathrm{I}, k_I \to \infty)$ for positive feedback and coherent FFL respectively). These expressions hold for independent regulation of a single gene by two species (no physical direct or indirect interaction between the two transcription factors), but the results discussed here are the same considering other types of logic production gates or additional cooperativity parameters.

In the numerical simulations, we fix the degradation rates (usually to unity) and equilibrium concentrations for each species (typically in the range $10^2 - 10^3$). Since we control for interactions with predefined susceptibilities, we recalculate, either analytically or numerically, the basal production rates α_X and activation/repression thresholds k_X to achieve the desired equilibrium concentrations and pairwise susceptibilities. Hill coefficients n are varied between 2 and 6 depending on the wanted output susceptibility s_O (the susceptibility is related to the *steepness* of an effective activation function [1, 2] and thus for $s_O = 5$, for instance, we should use $n \geq 6$).

Deterministic kinetic equations have been solved using Fortran codes and a fourth order Runge-Kutta algorithm. For the stochastic simulations, we employed Gillespie's algorithm [15]. Propensities are given by the production and degradation terms in Eqs. (6.70) (where n_X now represents number of molecules of species X) with a suitable rescaling of basal rates and activation/repression thresholds by a 'cell volume' factor V_X: $\alpha'_X \equiv \alpha_X \cdot V_X$ and $k'_X = k_X \cdot V_X$. The noise strength in each molecular species can thus be independently varied from the other components by its own volume factor. In the numerical simulations we change only the input signal noise keeping $V_S = V_O = 1$ (Fig. S5A).

The power spectral density has been numerically obtained by fast Fourier transform of the autocorrelation function (using FFT Matlab subroutines). We run large enough ensembles of stochastic trajectories with different initial conditions, $\sim 10,000$, to ensure convergence of the autocorrelations.

7 Model analysis from experimental data

In order to assess if the new detection properties discussed for feed–forward loops can be found in natural or synthetic genetic circuits, we analyzed three experimental works where simple mathematical models of incoherent and coherent FFLs can be fitted to experiments.

Kaplan et al. [25] have recently measured *in vivo* and at high resolution the production rate of the galETK operon for a wide range of concentrations of its two natural inducers, cAMP and galactose. Expression of GalE by these inputs is mediated by an incoherent FFL of type I (see Figure 7A), where the transcription factor CRP (activated by cAMP) activates both the galE gene and an intermediate transcription factor GalS, which is a repressor of GalE (whose activity is dependent on D-galactose). A second constitutive repressor, GalR, was removed from the original system. The GalE-$\Delta GalR$ production rate was fitted by Kaplan et al. to a Hill function dependent on cAMP and galactose concentrations [25]. We used this model to obtain the amplitude response (Figure 7B) and calculate the signal detection properties shown in Figure 7C. The dynamics of the incoherent FFL is given by the following equations:

$$\begin{aligned}
\frac{dC}{dt} &= \alpha_C \cdot [cAMP] - \delta \cdot C, \\
\frac{dG_S}{dt} &= \frac{C^{h1}}{1 + [galactose]/k_g} - \delta \cdot G_S, \\
\frac{dG_E}{dt} &= \frac{1}{1 + (G_S/k_{Gs})^{h2}} \cdot \frac{(C/k_C)^{h3}}{1 + (C/k_C)^{h3}} - \delta \cdot G_E,
\end{aligned} \tag{7.72}$$

where C is activated CRP, $G_S \equiv$ GalS and $G_E \equiv$ GalE. We assume that cAMP activates CRP in a linear fashion [25, 26]. Parameters showing a good fit to experimental data [25] are $k_C = k_{Gs} = k_g = 5$ mM, $h1 = h2 = h3 = 1$. For simplicity we assume that all protein degradation rates are given by cell growth and division, $\delta = \log(2)/\tau_{div} = 1$, which implies an average cell division time of $\sim$ 40 min, and $\alpha_C = \delta$, so that active CRP at equilibrium is equal to cAMP concentration. With these equations and parameters the GalE production rate (assuming CRP and GalS are at equilibrium) reproduce well the experimental input function of the galE-ΔGalR system, compare Figure 7B in main text and Figure

2 in Ref. [25]. The negative autoregulation of GalS has not been included in the model since it has little effect on the active repressor level and non-monotonicity of the input function [25].

To understand the peaked shape of the production rate as a function of cAMP levels (amplitude filter) we fix the galactose concentration and examine how global and pairwise susceptibilities change with cAMP, Figure S8. The susceptibilities for this simple model are easily calculated from Eq.(7.72) :

$$\begin{aligned} s_{SI} &= h1, \\ s_{OS} &= -h2\frac{(G_S/k_{Gs})^{h2}}{1+(G_S/k_{Gs})^{h2}}, \\ s_{OI} &= \frac{h3}{1+(C/k_C)^{h3}}, \end{aligned} \tag{7.73}$$

where the input $I \equiv$ CRP, sensor $S \equiv$ GalS and output $O \equiv$ GalE and species concentrations are at equilibrium. We see that the global susceptibility s_O (red line in Figure S8B) is positive and reaches its maximal values at the lowest cAMP levels, due to the contribution of the direct CRP/GalE interaction (black dashed line). The negative GalS/GalE susceptibility (green line) is very small at low cAMP concentrations since the amount of repressor activated by CRP is below threshold. Note that in Eqs. (7.73), $s_{OI} > 0$ but it has the typical form of a *repressor* Hill function, decreasing with CRP concentration due to saturation of the GalE promoter. The s_{OS} negative susceptibility, on the other hand, changes with GalS as an *activator* Hill function, and is maximal at high repressor levels. Therefore the global susceptibility decreases with cAMP and, when the inhibitory interaction dominates, becomes negative. The cAMP level of zero susceptibility corresponds to the maximum of the GalE production (asterisk and blue line in Figure S8A) since from this point GalE production starts to decrease.

This situation is independent of the specific models and interactions, and is expected for general incoherent FFLs showing an amplitude filter regime. As another instance, we analyze the model proposed by Basu et al. [27] which qualitatively reproduces the experimental response of a synthetic band-detect circuit to an external acyl-homoserine lactone (AHL) signal, Figure S9A. The inducer AHL activates the expression of lambda repressor (CI) and Lac repressor (LacI). LacI expression is inhibited by CI, effectively forming an incoherent FFL starting with AHL/LuxR (input) and ending at LacI (output). A GFP reporter under control of LacI repressor monitorizes the system response, Figure S9A. The dynamical equations provided by Basu et al. [27] are

$$\frac{dR}{dt} = \rho_R[LuxR]^2A^2 - \gamma_R R$$

$$
\begin{aligned}
\frac{dC}{dt} &= \frac{\alpha_C R^{\eta_3}}{\theta_R^{\eta_3} + R^{\eta_3}} - \gamma_C C \\
\frac{dL}{dt} &= \frac{\alpha_{L1}}{1 + (\frac{C}{\beta_C})^{\eta_2}} + \frac{\alpha_{L2} R^{\eta_3}}{\theta_R^{\eta_3} + R^{\eta_3}} - \gamma_L L \\
\frac{dG}{dt} &= \frac{\alpha_G}{1 + (\frac{L}{\beta_L})^{\eta_1}} - \gamma_G G
\end{aligned} \tag{7.74}
$$

where $A \equiv$ external AHL concentration, $R \equiv$ LuxR/AHL complex, $C \equiv$CI, $L \equiv$LacI and $G \equiv$GFP. Parameters are those fitting the experimental data of BD2 strain [27], $\alpha_{L1} = 1\mu M min^{-1}$, $\alpha_{L2} = 1\mu M min^{-1}$, $\alpha_C = 1\mu M min^{-1}$, $\alpha_G = 2\mu M min^{-1}$, $\beta_C = 0.008\mu M$, $\beta_L = 0.8\mu M$, $\theta_R = 0.01\mu M$, $\gamma_L = 0.0231 min^{-1}$, $\gamma_R = 0.0231 min^{-1}$, $\gamma_C = 0.0692 min^{-1}$, $\rho_R = 0.5\mu M^{-3} min^{-1}$, $LuxR = 0.05\mu M$, $\eta_1 = 2$, $\eta_2 = 2$ and $\eta_3 = 1$.

Similar to the galactose system, the GFP response behaves as an amplitude filter for external AHL concentration, Figure S9B. Around to the maximum, where the susceptibility of the output element of the incoherent FFL (LacI) is close to zero, Figure S9C, the system filters low frequency oscillations and noise (inset of Figure S9B). The high–pass frequency filter for an oscillatory AHL signal is illustrated in Figure S9D, compare with Figure 3F in main text.

Different sugar utilization genes in *E. coli* are induced by cAMP through a coherent FFL architecture [26]. The inducer cAMP, produced in the cell upon glucose starvation, activates the master regulator CRP. This controls the expression of intermediate transcription factors(sensors) which are activated by specific sugars and, together with CRP, regulate the response of several genes (outputs) involved in the metabolism of the corresponding sugar. As in the previous example for the galactose system, the production rate of both sensor and output genes has been experimentally measured for coherent FFLs controling *E. coli* response to four different sugar systems: arabinose, maltose, rhamnose and fucose. Of these, one of the simplest to analyze is the maltose utilization module, Figure 7B in main text. Production of the sensor species, MalT transcription factor, is only dependent upon cAMP/CRP and not on maltotriose [26]. However, MalT binds promoters of the malEFG, malPQ and malK operons as an oligomer stabilized by the inducer sugar [28, 29]. CRP and MalT both act cooperatively to activate MalE and MalK production [30]. With these facts in mind, we proposed a simple mathematical model of a coherent FFL based on Hill type regulation functions [24] to fit the experimental production rates of Kaplan et al. [26]. We specialized to the malEFG and malPQ operons, since MalK is known to directly interact with MalT in a negative feedback loop [28]. The equations for the CRP/MalT/MalE coherent FFL used are

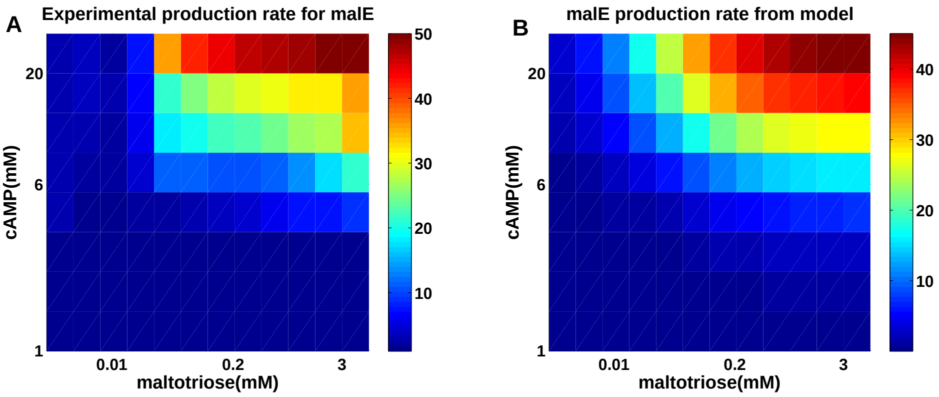

Figure 1: A. Experimental input function for the malEFG regulon as a function of external cAMP and maltotriose inducers (data from Kaplan et al. [26]). B. Production rate of MalE generated by the model in Eqs. (7.75). All molecular species concentrations are assumed to be at equilibrium.

$$\begin{aligned}
\frac{dC}{dt} &= \alpha_C \cdot cAMP - \delta \cdot C, \\
\frac{dM_T}{dt} &= \frac{\alpha_T + \beta(C/K_1)^{h1}}{1 + (C/K_1)^{h1}} - \delta \cdot M_T, \\
\frac{dM_E}{dt} &= \alpha_E \frac{s(C/K_2)^{h2}(M_T/K_3)^{h3}}{1 + s(C/K_2)^{h2}(M_T/K_3)^{h3}} - \delta \cdot M_E,
\end{aligned} \tag{7.75}$$

where s is the inducer sugar(maltotriose) C stands for active CRP, M_T for active MalT transcription factor and M_E for MalE product. We fitted independently the Mal_T and Mal_E experimental production rates to the mathematical form given by Eqs. (7.75) using a nonlinear optimization method with constraints (*fmincon* subroutine in Matlab 7.3) restricting the parameters to positive values. We took $\beta = 65$ mM and $\alpha_E = 50$ mM from the experimental values of MalT and MalE maximal production rates, and fitted the thresholds $K_1 = 5.6$, $K_2 = 7.6$, $K_3 = 7.1$ mM^{-1} and the Hill coefficients $h_1 = 1.5$, $h_2 = 2.4$, $h_3 = 1.3$ to the experimental data for MalT and MalE (root mean square errors 0.06 and 0.07 respectively, which are within the experimental 10% mean relative error [26]. We allowed for a small basal ($\alpha_T = 4.4$ mM) activation of MalT by CRP, and set the degradation rate by cell growth and division $\delta = 0.65 \cdot \log(2)$ h^{-1} as measured from the experimental average division rate [26]. The production rate of MalE for the model given by Eqs. (7.75) is compared to the experimental production rate obtained by Kaplan et al. [26] in Figure 1. The simple model proposed is

of course not unique, and different mathematical functions can be fitted to the experimental data. We also considered models where some MalE activation is possible either by CRP or MalT alone, and nonlinear posttrancriptional activation of MalT by the inducer sugar s. The quality of the fittings as measured by the root mean squared error(rmse), however, was the same than for the simple model ($0.06 < rmse < 0.08$) and the results for amplitude and frequency SNRs qualitatively similar to those shown in Figure 7E,F in main text.

References

[1] Paulsson J. (2004) *Nature* **427** 415-418.

[2] Paulsson J. (2005) *Physics of Life Reviews* **2** 157-175.

[3] Gomez-Uribe C., Verghese G. C. and Mirny L. A. (2007) *PLoS Comp. Biol.* **3** e246.

[4] Hornung G. and Barkai N. (2008) *PLoS Comp. Biol.* **4** e8.

[5] Samoilov M., Arkin A. and Ross J. (2002) *J. Phys. Chem. A* **106** 10205-10221.

[6] Hooshangi S., Thiberge S. and Weiss R. (2005) *Proc. Natl. Acad. Sci. USA* **102** 3581-3586.

[7] Gillespie D. T. (2000) *The Journal of Chemical Physics* **113** 297-306.

[8] Thattai M. and van Oudenaarden A. (2001) *Proc. Natl. Acad. Sci. USA* **98** 8614-8619.

[9] Tomioka R., Kimura H., Kobayashi T. J. and Aihara K. (2004) *J. Theor. Biol.* **229** 501-521.

[10] Keizer J, *Statistical Thermodynamics of Nonequilibrium Processes* (1987) Springer-Verlag.

[11] Elf J. and Ehrenberg M. (2003) *Genome Res.* **13** 2475-2484.

[12] van Kampen, N. G. (1992) *Stochastic processes in Physics and Chemistry* North-Holland, Amsterdam.

[13] Gardiner C. (1985) *Handbook of stochastic methods* Springer-Verlag, Berlin.

[14] Tanase-Nicola, S., Warren P. B. and ten Wolde P. R. (2006) *Phys. Rev. Lett.* **97** 068102.

[15] Gillespie D. T. (1977) *J. Phys. Chem.* **81** 2340–2360.

[16] Gajic Z *Linear Dynamic Systems and Signals* (2003) Prentice Hall, New Jersey, USA.

[17] Warren P., Tanase-Nicola, S. and ten Wolde P. R. (2007) *The Journal of Chemical Physics* **125** 144904.

[18] Shibata T. and Fujimoto K. (2005) *Proc, Natl. Acad. Sci. USA* **102** 331-336.

[19] Gammaitoni L., Hänggi P., Jung P. and Marchesoni F. (1998) *Reviews of Modern Physics* **70** 223-283.

[20] McNamara B. and Wiesenfeld K. (1989) *Physical Review A* **39** 4854-4869.

[21] Alon U. (2007) *Nature Reviews Genetics* **8** 450-461.

[22] Simpson M. L., Cox C. D. and Sayler G. S. (2003) *Proc. Natl. Acad. Sci. USA* **100** 4551-4556.

[23] Austin D W *et al.* (2006) *Nature* **439** 608-611.

[24] Alon U. *An Introduction to Systems Biology: Design Principles of Biological Circuits* (2007) Chapman & Hall, London, UK.

[25] Kaplan S., Bren A,. Dekel E. and Alon U. (2008) *Mol. Syst. Biol.* **4** 203-211.

[26] Kaplan S., Bren A., Zaslaver A., Dekel E. and Alon U. (2008) *Molecular Cell* **29** 786-792.

[27] Basu S, Gerchmann Y, Collins CH, Arnold FH, Weiss R (2005) *Nature* 434:1130-1134.

[28] Boos W and Böhm A (2000) *Trends in Genetics* **16** 404-409.

[29] Danot O (2001) *Proc. Natl. Acad. Sci. USA* **98** 435-440.

[30] Richet E (2000) *The EMBO Journal* **19** 5222-5232.

Table 1: Activation/repression functions for the production terms F_S and F_O

Network motif	F_S	F_O
Linear Cascade	$f_{act}(I, k_I)$	$f_{act}(S, k_S)$
Positive Feedback	$f_{act}(I, k_I) + f_{act}(O, k_O)$	$f_{act}(S, k_S)$
Negative Feedback	$f_{act}(I, k_I) \cdot f_{rep}(O, k_O)$	$f_{act}(S, k_S)$
Incoherent FFL	$f_{act}(I, k_I)$	$f_{rep}(I, k_I) \cdot f_{act}(S, k_S)$
Coherent FFL	$f_{act}(I, k_I)$	$f_{act}(I, k_I) + f_{act}(S, k_S)$
