# Supplementary material for: Trade-offs and Noise Tolerance in Signal Detection by Genetic Circuits
Source: PLoS One. 2010 Aug 26;5(8):e12314. doi: 10.1371/journal.pone.0012314 (PMC2928721; doi:10.1371/journal.pone.0012314)
Supplement: Table S1 — Definition of frequency detection parameters and corresponding susceptibility ranges for simple network motifs. (0.03 MB PDF) [file pone.0012314.s011.pdf]

Table S1: Definition of frequency detection parameters and corresponding susceptibility ranges for simple network motifs.

Network motif	*Frequency parameter*	*Maximum susceptibility*	*Minimum susceptibility*
Negative feedback	Feedback strength(FS) [1] $FS \equiv \lvert s_{OS} \cdot s_{SO} \rvert$	$s_O^{max} = \frac{smax \cdot s^*_{SI}}{1+FS}$	$s_O^{min} = \max\left(\frac{FS \cdot s^*_{SI}}{smax(1+FS)}, \frac{smin \cdot s^*_{SI}}{1+FS}\right)$
Positive feedback	Feedback strength(FS) [2] $FS \equiv \lvert s_{OS} \cdot s_{SO} \rvert$	$s_O^{max} = \frac{smax \cdot s^*_{SI}}{1-FS}$	$s_O^{min} = \max\left(\frac{FS \cdot s^*_{SI}}{smax(1-FS)}, \frac{smin \cdot s^*_{SI}}{1-FS}\right)$
Negative autoregulation on output	Autoregulation strength(ARS) [3] $ARS \equiv \lvert H_{OO} - 1 \rvert$	$s_O^{max} = \frac{smax \cdot s^*_{SI}}{1+ARS}$	$s_O^{min} = \frac{smin \cdot s^*_{SI}}{1+ARS}$
Positive autoregulation on output	Autoregulation strength(ARS) $ARS \equiv \lvert H_{OO} - 1 \rvert$	$s_O^{max} = \frac{smax \cdot s^*_{SI}}{1-ARS}$	$s_O^{min} = \frac{smin \cdot s^*_{SI}}{1-ARS}$
Incoherent FFL [4]	Relative strength(RS) $RS \equiv \left\lvert \frac{s_{OI}}{s_O} \right\rvert$	$s_O^{max} = s_{OI} + smax \cdot s^*_{SI}$ [5] $s_O^{max} = \frac{smax}{RS}$ [6]	$s_O^{min} = \frac{smin \cdot s^*_{SI}}{1+RS}$
Coherent FFL [7]	Relative strength(RS) $RS \equiv \left\lvert \frac{s_{OI}}{s_O} \right\rvert$	$s_O^{max} = s_{OI} + smax \cdot s^*_{SI}$ [8] $s_O^{max} = \frac{smax}{RS}$ [9]	$s_O^{min} = \frac{smin \cdot s^*_{SI}}{1-RS}$

[1] Maximum FS=$smax \cdot smax$.
[2] Maximum FS=1, otherwise the output steady state is unstable.
[3] For no autoregulation, $H_{OO} = 1$. For positive autoregulation, $0 < H_{OO} < 1$. For negative autoregulation, $H_{OO} > 1$.
[4] Here we analyze the type 3 Incoherent FFL, $s_{OI} < 0$, with input/sensor and sensor/output interactions positive as in the rest of the circuits.
[5] For $RS < \frac{1}{s^*_{SI}-1}$, $s_{OS} = smax$ and s_O^{max} decreases as s_{OI} changes in the interval $[0, -smax]$.
[6] For $RS > \frac{1}{s^*_{SI}-1}$, $s_{OI} = -smax$ and s_O^{max} decreases as s_{OS} changes in the interval $[smax, 0]$.
[7] Here we analyze the type I Coherent FFL, $s_{OI} > 0$ with input/sensor and sensor/output interactions positive.
[8] For $RS < \frac{1}{1+s^*_{SI}}$, $s_{OS} = smax$ and s_O^{max} increases as s_{OI} changes in the interval $[0, smax]$.
[9] For $RS > \frac{1}{1+s^*_{SI}}$, $s_{OI} = smax$ and s_O^{max} decreases as s_{OS} changes in the interval $[smax, 0]$.
